# Quantitative risk assessment for the introduction of bluetongue virus into mainland Europe by long‐distance wind dispersal of *Culicoides* spp.: A case study from Sardinia

**DOI:** 10.1111/risa.14345

**Published:** 2024-07-02

**Authors:** Amandine Bibard, Davide Martinetti, Aymeric Giraud, Albert Picado, Karine Chalvet‐Monfray, Thibaud Porphyre

**Affiliations:** ^1^ Global Innovation Boehringer Ingelheim Animal Health France Saint‐Priest France; ^2^ Laboratoire de Biométrie et Biologie Évolutive, UMR 5558 Université Claude Bernard Lyon 1, CNRS, VetAgro Sup Villeurbanne France; ^3^ Epidémiologie Des Maladies Animales et Zoonotiques, UMR EPIA Université Clermont Auvergne, INRAE, VetAgro Sup Saint‐Genès‐Champanelle France; ^4^ Biostatistique et Processus Spatiaux, UMR 0546 INRAE Avignon France

**Keywords:** bluetongue virus, HYbrid Single‐Particle Lagrangian Integrated Trajectory, midges, orbivirus, risk

## Abstract

Europe faces regular introductions and reintroductions of bluetongue virus (BTV) serotypes, most recently exemplified by the incursion of serotype 3 in the Netherlands. Although the long‐distance wind dispersal of the disease vector, *Culicoides* spp., is recognized as a virus introduction pathway, it remains understudied in risk assessments. A Quantitative Risk Assessment framework was developed to estimate the risk of BTV‐3 incursion into mainland Europe from Sardinia, where the virus has been present since 2018. We used an atmospheric transport model (HYbrid Single‐Particle Lagrangian Integrated Trajectory) to infer the probability of airborne dispersion of the insect vector. Epidemiological disease parameters quantified the virus prevalence in vector population in Sardinia and its potential first transmission after introduction in a new area. When assuming a 24h maximal flight duration, the risk of BTV introduction from Sardinia is limited to the Mediterranean Basin, mainly affecting the southwestern area of the Italian Peninsula, Sicily, Malta, and Corsica. The risk extends to the northern and central parts of Italy, Balearic archipelago, and mainland France and Spain, mostly when maximal flight duration is longer than 24h. Additional knowledge on vector flight conditions and Obsoletus complex‐specific parameters could improve the robustness of the model. Providing both spatial and temporal insights into BTV introduction risks, our framework is a key tool to guide global surveillance and preparedness against epizootics.

## 1 INTRODUCTION

Bluetongue disease is recognized as a global veterinary concern due to its economical and animal health impact. It affects both domestic and wild ruminants, and it is mostly transmitted by small flying Diptera (midges) of the genus *Culicoides*. In Europe, since the bluetongue virus (BTV) serotype 10 (BTV‐10) first emerged in Portugal and Spain in 1956–1960, 12 additional viral serotypes among the 36 identified nowadays (Ries et al., 2021) have been reported in the continent (BTV‐1, ‐2, ‐3, ‐4, ‐6, ‐8, ‐9, ‐11, ‐14, ‐16, ‐25, ‐27) (Saminathan et al., 2020). BTV‐3 first emerged in Sicily (2017), then in Sardinia (2018), and very recently in September 2023, in the Netherlands (Holwerda et al., 2023; Wageningen Bioveterinary Research [WBVR], 2023). In Italy, BTV‐3 was reported to affect mainly small ruminants with mild clinical signs and remained mostly subclinical in cattle (Cappai et al., 2019). In Sicily, no new clinical cases have been reported in sheep since 2017, but in 2018, 2019, and 2022, one single cattle was diagnosed as BTV‐3 positive each year (Bollettino Epidemiologico Nazionale Veterinario (izs.it)). In Sardinia, the virus has been reported circulating every year since 2018 but the infected area has not so far exceeded the endemic zone in the southern part of the island (WAHIS (woah.org)). In contrast, the BTV‐3 strain circulating in the Netherlands has been associated with severe clinical signs, including deaths, in both sheep and cattle and has spread very rapidly beyond the initial index sites (Holwerda et al., 2023). A pathogenic viral strain of BTV‐3 has also previously been reported in Israel (Golender et al., 2020).

Several routes of BTV introduction have been imputed solely or in combination: import of live animals through legal or illegal trade, import of germplasm (semen and/or embryos), movement of wild animals, airborne dispersion of vectors, import of adult vectors by aircraft or use of poorly attenuated modified live vaccines (Gale et al., 2015; Hartley et al., 2013; Hoar et al., 2004; Mintiens et al., 2008; Napp et al., 2011, 2013; Simons et al., 2019). Although the route of introduction into the Netherlands is still under investigation, the suspected route of BTV‐3 introduction into Sicily and Sardinia was the long‐distance wind dispersal (LDWD) of infected vectors from Tunisia (Aguilar‐Vega et al., 2019; Cappai et al., 2019). The BTV‐3 circulation in Sardinia, on the doorstep of mainland Europe, poses a real threat to surrounding free countries in Europe. In this study, we investigated which European countries could be at risk of BTV‐3 introductions from Sardinia as a direct consequence of LDWD of infected vectors.

Quantitative Risk Assessment (QRA) frameworks aim at describing the sources of risk and quantifying risk levels and their impact. In the last 15 years, their use has significantly increased and has been improved thanks to methodological guidelines (Vos et al., 2011; World Organisation for Animal Health [WOAH], 2010) and computational capacities. Specifically for BTV, introduction risk assessments were developed mostly at country or regional level to enhance national surveillance in high‐risk regions or to define high‐risk periods of potential introduction (Gale et al., 2015; Gubbins et al., 2010; Hartley et al., 2013; Hoar et al., 2004; Maurella et al., 2019; Napp et al., 2013; Nelson et al., 2022; Roberts et al., 1993; Sagüés et al., 2019; Simons et al., 2019; Sutmoller & Wrathall, 1997). Recently, a generic risk framework at European scale has been developed for BTV considering different pathways of introduction including the long‐distance wind dispersion of vectors (Simons et al., 2019). Nonetheless, the source of infection in that study was arbitrary defined as a buffer zone of 300 km around each country without considering variations in topography, wind direction, speed, and vector survival conditions. On the other hand, evidence of long‐distance wind dispersion of *Culicoides* spp. has been shown for up to 700 and 500 km over sea (Ducheyne et al., 2011; Eagles et al., 2012, 2014) and over land (García‐Lastra et al., 2012) respectively, under suitable conditions. The LDWD of midges depends on meteorological conditions, and these need to be considered in the analyses.

Nowadays, meteorological data are collected worldwide at high resolution, and atmospheric dispersion models exist to simulate trajectories of particles through the atmosphere, mimicking the wind‐mediated passive flight of insects. Such meteorological models were previously used to retrospectively identify the potential source of new virus introductions, such as the BTV‐2 and BTV‐7 introductions into Australia from Oceania islands (Eagles et al., 2014), the BTV‐3 introduction into Sicily from Tunisia (Aguilar‐Vega et al., 2019), and *Culicoides imicola* migration to mainland France from Corsica (Jacquet et al., 2016). We hypothesize that such an atmospheric dispersion model could suitably infer the probability of long‐distance *Culicoides* dispersion from a known source to any destination in Europe and could therefore inform a QRA framework at European scale.

In this study, we have estimated the risk of BTV incursion into mainland Europe via LDWD from Sardinia and identified key parameters to this risk. To do so, we developed a QRA framework considering the complex vector–host–environment interactions of BTV, with multiple host and vector species, and applied this framework to the risk of BTV‐3 introduction from the southwestern parts of Sardinia. From our results, high‐risk areas and high‐risk periods of BTV‐3 introduction into European free areas were identified, providing useful guidance for surveillance.

## 2 MATERIALS AND METHODS

### 2.1 Framework overview

**FIGURE 1:**
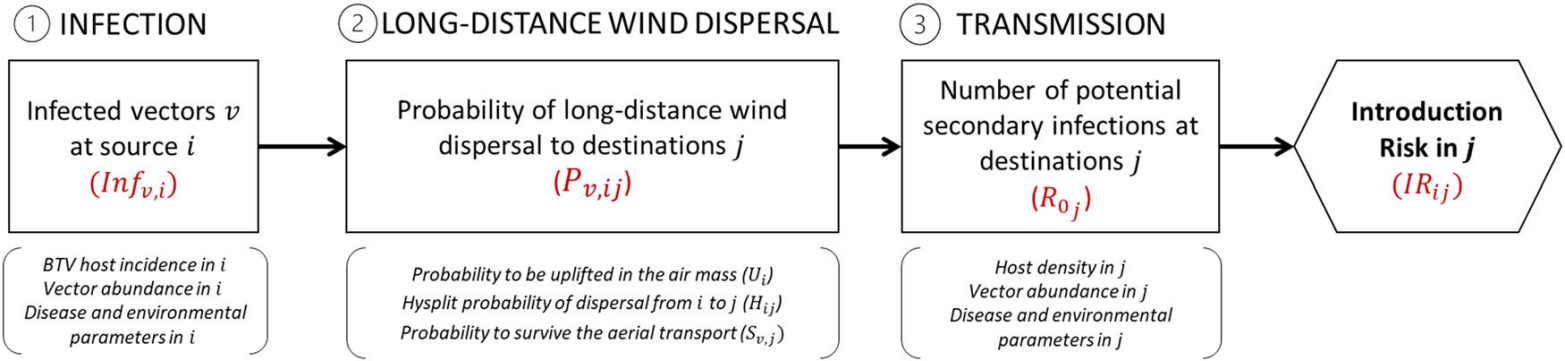
Risk Assessment (RA) framework quantifying the risk of bluetongue virus introduction from a source area i to a destination area by long‐distance wind dispersal (LDWD) of an infected *Culicoides* vector of species v. Introduction risk calculation at destination j ($IR_{ij}$) is a combination of three steps.

A QRA framework (Figure 1) was developed based on three evaluation steps:

1. $Inf_{v,i}:\;$ The level of infection in each vector species v at the source i, that is, the number of vectors of species v that are infected (and therefore infectious) within the infection source area i;
2. $P_{v,ij}:$ The probability of LDWD of living vectors of species v from source i to any destination j in Europe;
3. $R_{0j}$ : The potential of onward virus transmission at destination j, that is, the geometric average rate of secondary infections generated by one primary vector infection at destination j.

Our model framework considers two susceptible host species h (“C” for cattle and “S” for small ruminants, the latter comprising sheep and goats), two competent vector species v (“1” for *C. imicola* and “2” for Obsoletus complex, the latter comprising *Culicoides obsoletus* and *Culicoides* *scoticus* species) and the environmental conditions both at the source i and destination j.

We estimated the risk of a new BTV introduction into any destination 𝑗 ($IR_{ij}$) in Europe from source i, by computing outputs from the three evaluation steps, as follows:

$$IR_{\mathrm{ij}}\;=\;\sum_{v\in \left\{1\;,2\right\}}\left(\mathrm{In}f_{v,i}\;\times \;P_{v,\mathrm{ij}}\right)\;\times \;R_{0j}.\;$$

The value $IR_{ij}$ should be interpreted as a daily rate of new infections generated by both vector species v at destination j for each week, and considering the total number of infectious vectors that could be dispersed by the wind from i to j for each week as the sum of each vector species v. Here, $IR_{\mathrm{ij}}$ > 1 indicates that a potential introduction of the virus at destination j may originate from source i and may be established in the local population, whereas $IR_{\mathrm{ij}}$< 1 indicates that no introduction of the virus into destination *j* may occur. The number of weeks with a risk greater than 1 ($W_{IR>1}$) and the mean of IR over the entire study period ($\bar{IR}$) were also calculated for each destination j and mapped to evidence high‐risk regions.

The study period was limited to the 36 weeks from week 11 (mid‐March) to week 46 (mid‐November), assuming low outdoor *Culicoides* activity during the winter period (Villard et al., 2019). The study grid coordinates were set between longitudes 13°W and 37°E, and latitudes 28°S and 71°N. All datasets were aggregated at the final resolution of 0.5° (grid cell dimensions of approximately 50 × 50 km^2^) to compute the model. A minimal threshold of 1 vector and 1 host per km^2^ (2500 individuals per population—vector or host—and per each grid cell) was set to compute the model, assuming that BTV transmission is unlikely in areas with too low numbers of host or vector. We decided to use a lower threshold compared to other studies (1 vs. 10 per km^2^) (Hartemink et al., 2009) because of the high dimension of our grid and in order not to be more conservative.

Table 1 summarizes the model input parameters, their description, and data sources. Details on equations and database computations are provided in Supplement Information S1. Data aggregation and model computation were performed using R Statistical Software v4.2.2 (R Core Team, 2022) and required specific packages, such as “sf” (Pebesma, 2022), “terra” (Hijmans, 2023), “tidyterra” (Hernangómez, 2023), “rnaturalearth” (Massicotte & South, 2023), and “exactextractr” (Baston, 2022).

**TABLE 1:** Description, formulas, and sources of input parameters used in the risk assessment model of bluetongue virus (BTV) infected *Culicoides* spp. by long‐distance wind dispersal (LDWD).

Var	Description	Comments and formula	Source/Reference
h	Host population type, C (cattle) or S (small ruminants)		–
v	Vector population type, 1 (*Culicoides imicola*) or 2 (Obsoletus complex)		–
$N_{h}$	Host population species h	Minimal threshold of 1 animal per km^2^ was set	Livestock GLW4 (Gilbert et al., 2018)
$\mathrm{Pre}v_{h}$	Disease prevalence in source area in hosts species h	$\mathrm{Pre}v_{h}=\mathrm{number}\;\mathrm{of}\;\mathrm{cases}/N_{h}$	(IZST, 2022)
$N_{v}$	Maximal vector abundance recorded in a year per trap of species v	Minimal threshold of 1 vector per km^2^ was set	VectorNet Data Series3 (Balenghien et al., 2020)
w	Probability function of vector presence, such as $wN_{v},\;$ is the expected vector abundance of species v for each week	Depend on temperature, humidity, and elevation	(Conte et al., 2020), Supplement Information S1
$Inf_{v,i}$	Number of infected vector of species v at source i	Equation detailed in main text	(Turner, Bowers & Baylis, 2013), Supplement Information S1
$U_{i}$	Probability to be uplifted in the air mass	Constant of 0.25	–
$H_{ij}$	Probability of wind dispersal from source i to destination j inferred from HYSPLIT simulations	Mean frequency of deposition in each destination cell over the total forward simulations run by HYSPLIT model from source i, averaged for each week over the 3 years 2020–2022	(Stein et al., 2015)
$a_{v}$	Biting rate of vector species v	$a_{1}\left(T\right)=\;0.00014\;\times \;T\;\times \;\left(T\;-\;3.6966\;\right)\;\times \;{\left(41.8699\;-\;T\;\right)}^{\frac{1}{2.7056}}\;\mathrm{for}\;T>3.7^{\circ }C$ $a_{2}\left(T\right)=\;0.000171\;\times \;T\;\times \;\left(T\;-\;3.6966\;\right)\;\times \;{\left(41.8699\;-\;T\;\right)}^{\frac{1}{2.7056}}\;\mathrm{for}\;T>3.7^{\circ }C$	(Aguilar‐Vega, Bosch, Fernandez‐Carrion, Lucientes, & Sanchez‐Vizcaino, 2020; Mullens, Gerry, Lysyk & Schmidtmann, 2004)
$\omega_{v}$	Virogenesis rate (i.e., rate at which latent vector species v become infectious = 1/extrinsic incubation period in days)	$\omega_{1}\left(T\right)=\;0.016\;\times \;\left(T\;-\;12.7\right)\;\mathrm{for}\;T>12.7^{\circ }C$ $\omega_{2}\left(T\right)=\;0.0003\;\times \;T\;\times \;\left(T\;-\;10.4057\right)\;\mathrm{for}\;T>10.4^{\circ }C$	$\omega_{1}$ (Sanders et al., 2011) $\omega_{2}$ (Mullens et al. 2004; Turner et al., 2013)
$\mu_{v}$	Natural mortality rate of vector species v	$\mu_{1}\left(T\right)=\;1-\;\left(1e-5\;\times \;T^{3}-\;0.001\;\times \;T^{2}+\;0.0187\;\times \;T\;+\;0.8924\right)\mathrm{for}\;T>10^{\circ }C$ $\mu_{2}\left(T\right)=\;0.015\;\times \;\exp\;\left(0.063\;\times \;T\right)\;\mathrm{for}\;T>0^{\circ }C$	$\mu_{1}$ (White, Sanders, Shortall, & Purse, 2017) $\mu_{2}$ (Wittmann, Mellor, & Baylis, 2002)
$\beta_{v}$	Probability of transmission from host to vector of species v	$\beta_{1}\left(T\right)=\;0.0003699\exp\;\left(0.1725\;\times \;T\right)$ $\beta_{2}\left(T\right)=\;0.005465\;\exp\;\left(0.159\;\times \;T\right)$	(Turner et al., 2013)
$m_{hv}$	Ratio of vectors species v to hosts species h	$m_{vh}=wN_{v}/N_{h}$	–
$\phi_{hv}$	Proportion of vectors species v attracted to hosts species h	$\phi_{Cv}\;=m_{Sv}/\left(m_{Sv}+\;\sigma_{v}\;\times \;m_{Cv}\right)$ $\phi_{Sv}\;=\;1\;-\phi_{Cv}\;$ $\phi_{h1}\;=\;1\;-\;\phi_{h2}\;$	–
$\sigma_{v}$	Host preference for vector species v	$\sigma_{1}\;=\;\sigma_{2}\;=\;0.15$ ($\sigma_{v}$<1 indicates a preference for cattle)	(Aguilar‐Vega et al., 2020)
$r_{h}$	Recovery rate (1/ duration of viremia in days) in host species h	$r_{C}=\frac{1}{29}$ $r_{S}=1/20$	(Dorea et al., 2017)
$d_{h}\;$	Disease induced mortality in host h	$d_{C}=0$ $d_{S}=0.0078\;$	$d_{C}$ (Guis et al., 2012)$d_{S}$ (Aguilar‐Vega et al., 2020)
b	Probability of transmission from vector to host	b=0.9	(Aguilar‐Vega et al., 2020; Hartemink et al., 2009)

*Note*: Rates are given per day.

Abbreviation: HYSPLIT, HYbrid Single‐Particle Lagrangian Integrated Trajectory.

#### 2.1.1 BTV infection at source (step 1)

**FIGURE 2:**
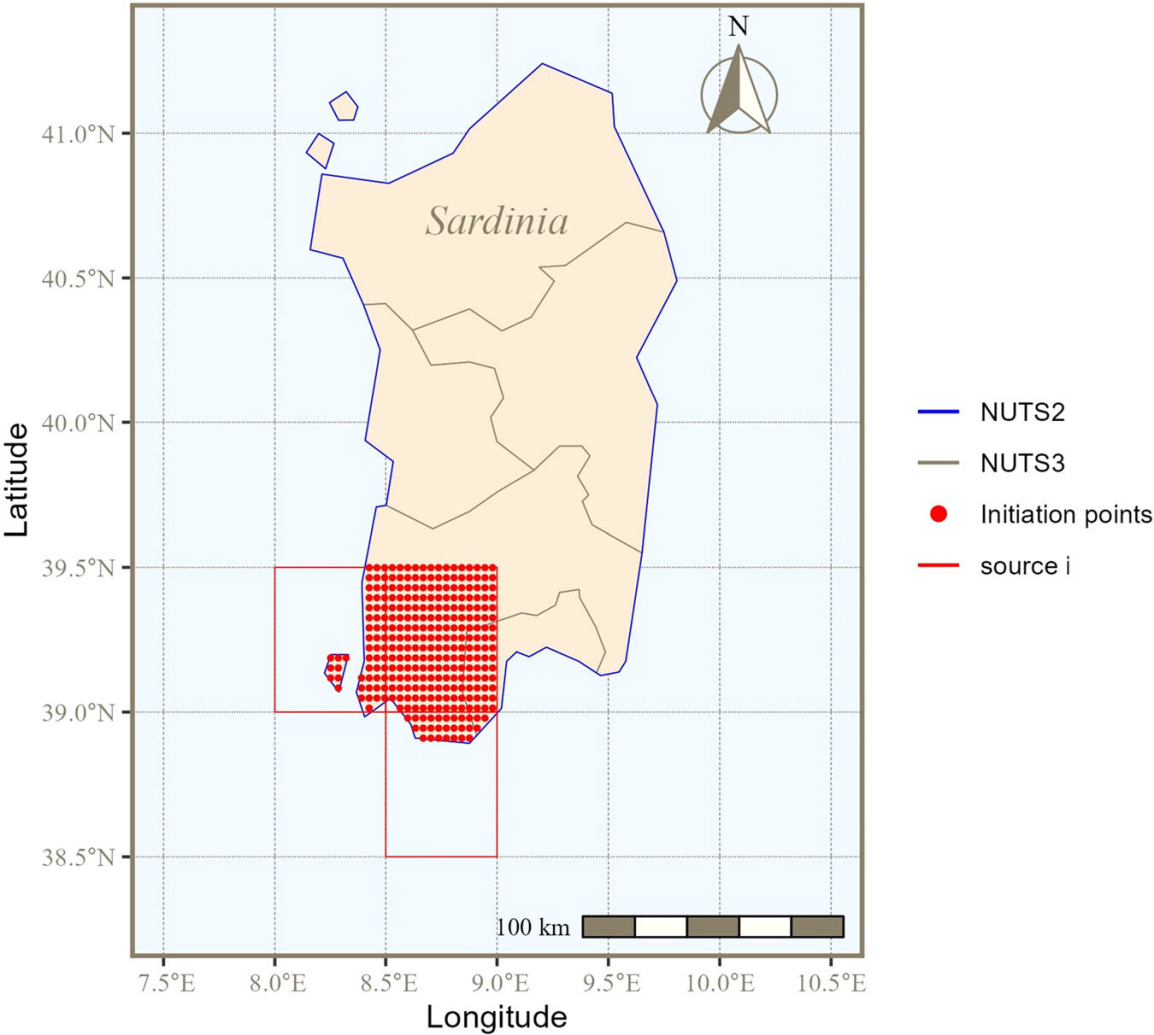
Source area i was defined in the southwest of Sardinia. Atmospheric simulations in HYbrid Single‐Particle Lagrangian Integrated Trajectory (HYSPLIT) were initiated from 290 locations located on land surface of this area.

The source area i was defined as the southwestern part of Sardinia where BTV‐3 outbreaks have been reported (Cappai et al., 2019) (Figure 2). The disease was considered at endemic equilibrium, that is, with a balanced number of midges transitioning among the three infection stages—susceptible, latent, and infectious—over time.

The number of susceptible midges was approximated by the daily vector abundance in source i. We calculated a daily vector abundance in a given week of species v in source i, $w\cdot N_{v,i}$, where$\;N_{v,i}$ is the maximal abundance reported within a given year in source i and extracted from the VectorNet (Balenghien et al., 2020) data (maximal number per trap per day); and w is a probability function of midge presence (ranging between 0 and 1) depending on environmental conditions of the given week (Conte et al., 2009) to account for seasonal variation of the abundance (see Section S1.2).

The number of infected vectors (i.e., the sum of latent and infectious vectors) of species v in $(Inf_{v,\;i}$) was then expressed according to the number of susceptible, as follows (see Section S1.1):

(1) $$\mathrm{In}f_{v,i}=w\cdot N_{v,i}\;\frac{1}{\mu_{v}}\sum_{h\in \left\{C,S\right\}}\beta_{v}a_{v}\left(\phi_{\mathrm{vh}}\mathrm{pre}v_{h}\right),$$

where $w\cdot N_{v,i}$ is the expected daily number of susceptible vectors in the area i in a given week; 𝜇_𝑣_ the mortality rate of vector of species v; 𝛽*_𝑣_* the probability of transmission from a host species *ℎ* to a vector of species *𝑣* given an effective contact; *𝑎_𝑣_* the biting rate of vector species *𝑣*; *Φ𝑣ℎ* the proportion of vectors species *𝑣* attracted by hosts species *ℎ*; and 𝑝𝑟𝑒𝑣*_ℎ_* the proportion of infectious host species *ℎ* in area *𝑖* (disease prevalence in host).

The number of infectious hosts in the source i was set to 50 cases for cattle, and 200 cases for small ruminant, approximated from the number of BTV‐3 events reported by the official surveillance body of Sardinia in December 2022 (Istituto_Zooprofilattico_Sperimentale_della_Sardegna, 2022). The disease prevalence in i for each host species $h\;(\mathrm{pre}v_{h})$ was calculated by dividing the number of cases by the respective livestock population retrieved in source i from the Livestock Grid database (Gilbert et al., 2018) and was considered constant over the study period (Section S1.3 and Figure S3.2). Details on data sources and computation are provided in Section S1.3.

#### 2.1.2 Long‐distance wind dispersal (step 2)

Step 2 consisted of estimating the probability of LDWD from source to destination ($P_{v,ij}$), which is the combination of the probabilities to be uplifted in the windstream ($U_{i}$), to be subsequently transported by the wind to a destination area *𝑗* ($H_{ij}$), and to survive along the aerial transport until j ($S_{v,j}$), as follows:

(2) $$P_{v,ij}=U_{i}\times H_{ij}\times S_{v,j,}$$

where $U_{i}$ estimated the proportion of vectors that will be uplifted in the wind stream in average per week and subsequently long‐distance transported. In absence of reference in the literature, we estimated this proportion as fixed (0.25) and corresponding to the active proportion of midges flying at sunset and sunrise (see the following paragraph on conditions for initiation of atmospheric simulations).

An atmospheric dispersion model, HYSPLIT (Stein et al., 2015) (HYbrid Single‐Particle Lagrangian Integrated Trajectory), was used to infer $H_{ij}$. Forward simulation trajectories were initiated from 290 starting points comprised within land surface of the source area i, which covers approximatively 3300 km^2^ (resolution 0.5°) (Figure 2).

Trajectory simulations were run daily over the 36 weeks of the study period. In each day, wind trajectories simulations were initiated from nine timepoints corresponding to periods of flight activity of vectors (Elbers et al., 2016): around dusk (at sunset and 1 h before and after) and at dawn (at sunrise and in the following 5 h). Trajectories were set to start at a minimal altitude of 50 m above ground level (a.g.l.). Along each simulated trajectory, meteorological and atmospheric variables were recorded hourly, including air mass temperature and thickness of the planetary boundary layer (PBL). Each hourly checkpoint was deemed a viable destination location for the vector if the temperature was greater than 10°C (Braverman & Chechik, 1996; Sellers & Maarouf, 1991) and within the PBL; otherwise, further checkpoints of the trajectory were discarded. Trajectories were stopped based on two separate flight durations (dispersion): 24 and 48 h.

Finally, $H_{ij}$ was estimated by dividing the number of times per week the destination *𝑗* was reached by the total number of destinations from all simulations initiated from i during this week. Simulations were run from week 11 to week 46 (calendar weeks), independently for 2020, 2021, and 2022, and the final $H_{ij}$ value represents the average over the years for each calendar week.

The duration of passive flight in our scenarios (24 or 48 h) was considered not negligible as compared to the lifespan of the vector, and the probability to be still alive at destination j, $S_{v,j}$, was expressed as follows:

(3) $$S_{v,j}=e^{-\mu_{v,j}\;\times \;\mathrm{time}}$$

With time as the two scenarios of dispersion in days (24 or 48 h) and $\mu_{v,j}$ the mortality rate of vector species v calculated with environmental conditions in destination j.

For the sake of clarity in the presentation of results, $P_{v,ij}$ was only presented considering the conditions of Obsoletus complex and in this case was named $P_{ij}$. The expected probability of LDWD ($P_{ij}$) was computed for each given week of the study period and considering environmental conditions constant over the week. Furthermore, we computed the overall probability of LDWD (${\bar{P}}_{ij}$) as the mean of $P_{ij}$ over the whole study period. Both the expected probability $P_{ij}$ and the overall probability ${\bar{P}}_{ij}$ were then binned into four intervals (]0, 10^−4^]; ]10^−4^, 10^−3^]; ]10^−3^, 10^−2^]; ]10^−2^, 10^−1^]) to map four dispersion rings around the source area.

#### 2.1.3 BTV transmission at destination (step 3)

Disease transmissibility in each destination cell j was estimated through the ${R_{0}}_{j}$ (basic reproduction number), usually defined as the expected number of secondary cases produced, in a completely susceptible population, by a typical infectious individual (Driessche & Watmough, 2002). We used the $R_{0}$ formula defined for BTV by Turner et al. (2013) considering two‐host and two‐vector populations v, with ∈{1, 2} for *C. imicola* and Obsoletus complex and ℎ∈{C, S} for cattle and small ruminant populations, respectively. It combines four $R_{xy}$ components, with x defining the vector species at destination j that get infected due to one primary infectious vector of species y incoming from source i, such as

(4) $${R_{0}}_{j}=\sqrt{\frac{1}{2}\left[\left(R_{11}+R_{22}\right)+\sqrt{{\left(R_{11}+R_{22}\right)}^{2}-4\left(R_{11}R_{22}-R_{12}R_{21}\right)}\right]},$$

with

$$\;R_{11}=\left(\frac{\beta_{1}ba_{1}^{2}}{\mu_{1}}\right)\;\left(\frac{\omega_{1}}{\omega_{1}+\mu_{1}}\right)\left(\frac{m_{c1}\phi_{1}^{2}}{r_{c}+d_{c}}+\frac{m_{s1}{\left(1-\phi_{1}\right)}^{2}}{r_{s}+d_{s}}\right),$$

$$\;R_{22}=\left(\frac{\beta_{2}ba_{2}^{2}}{\mu_{2}}\right)\;\left(\frac{\omega_{2}}{\omega_{2}+\mu_{2}}\right)\left(\frac{m_{c2}\phi_{2}^{2}}{r_{c}+d_{c}}+\frac{m_{s2}{\left(1-\phi_{2}\right)}^{2}}{r_{s}+d_{s}}\right),$$

$$\begin{array}{cc} \;R_{12} & =\left(\frac{\beta_{1}ba_{2}a_{1}}{\mu_{2}}\right)\;\left(\frac{\omega_{2}}{\omega_{2}+\mu_{2}}\right)\\ & \left(\frac{m_{c1}\phi_{2}\phi_{1}}{r_{c}+d_{c}}+\frac{m_{s1}\left(1-\phi_{1}\right)\left(1-\phi_{2}\right)}{r_{s}+d_{s}}\right), \end{array}$$

$$\begin{array}{cc} \;R_{21} & =\left(\frac{\beta_{2}ba_{2}a_{1}}{\mu_{1}}\right)\;\left(\frac{\omega_{1}}{\omega_{1}+\mu_{1}}\right)\\ & \left(\frac{m_{c2}\phi_{2}\phi_{1}}{r_{c}+d_{c}}+\frac{m_{s2}\left(1-\phi_{1}\right)\left(1-\phi_{2}\right)}{r_{s}+d_{s}}\right), \end{array}$$

where $R_{11}$ is the average number of infectious *C. imicola* produced at destination by one infectious *C. imicola* incoming from source; $R_{22}$ the average number of infectious Obsoletus complex midges produced at destination by one infectious Obsoletus complex midge incoming from source; $R_{12}$ is the average number of infectious *C. imicola* produced at destination by one infectious Obsoletus complex incoming from source; and $R_{21}$ is the average number of infectious Obsoletus complex produced at destination by one infectious *C. imicola* incoming from source. For simplicity, the index *𝑗* of each $R_{xy}$ of ${R_{0}}_{j}\;$ was not expressed but is implicit.

It is worth noting that ${R_{0}}_{j}$ is actually the square root of the “Type Reproduction Number” $R_{T}$ defined for vector‐borne disease (Yakob & Clements, 2013). The ${R_{0}}_{j}$ is equivalent to the average number (geometric mean) of secondary infections at destination produced by one single infection. As such, an estimate of 𝑅_0__𝑗_ < 1 implies that disease transmission would not be sufficient for BTV to sustain in these conditions, whereas BTV would spread where 𝑅_0__𝑗_ > 1. The terms $\sqrt{R_{11}}$ and $\sqrt{R_{22}}$ represent the case where BTV would be transmitted by only one‐vector species, *C. imicola* or the Obsoletus complex, respectively. All infected vectors originating from i and surviving movement to j were considered infectious in j, and all local hosts in j were considered susceptible to the disease. Spatial distributions of host populations in Europe as considered in our study were extracted from the Gridded Livestock of the World (GLW3) (Gilbert et al., 2018) and are shown in Figure S3.2.

Input parameters involved in Equation (4) are described in Table 1. Parameters specific to the vector species v, like the virogenesis rate $\left(\omega_{v}\right)$, the mortality rate ($\mu_{v}$), or the probability of virus transmission from a host to vector ($\beta_{v}$), are dependent on the temperature, and the weekly mean in each destination cell j was used. The probability of virus transmission from a vector to a host (b) was considered constant, irrespective of the host and vector species involved (Aguilar‐Vega et al., 2020; Hartemink et al., 2009). The host recovery rates ($r_{h}$), defined as the inverse of the viremic period in host, were retrieved from a meta‐analysis performed by EFSA (Dórea et al., 2017). The median end day of viremia detection (by PCR or virus isolation) was taken as reference for each host species (29 days for cattle and 20 days for sheep and goats; assuming equal parameters for small ruminants). Disease mortality rates in hosts ($\mu_{h}$) are highly dependent of the species susceptibility and the viral serotype but were assumed to be low (0.0078) in small ruminants and even null in cattle (asymptomatic) (Maclachlan, 2011; Saminathan et al., 2020).

### 2.2 Sensitivity analysis

A global sensitivity analysis was performed to identify the most influential parameters of the model. We used the Sobol’ Indices method (Saltelli et al., 2010) which is a variance‐based statistical method and does not require a priori assumptions (linearity or additivity) of model behavior. First, maximal range domains for each of the 18 parameters tested were defined according to literature, and uniform distributions were applied for all factors (Supplement Information S2). Maximal flight duration and host prevalence were fixed at 24 h and 0.01, respectively. Second, the Sobol indices were estimated using the revised method of Jansen and Sobol (Saltelli et al., 2010), following 4‐step procedure: (1) two Latin hypercube samplings of each factor with 500,000 iterations, (2) computation of sampled values leading to 10^7^ sets of parameters combinations, (3) model run for each set of parameters combinations, and (4) calculation of the Sobol sensitivity indices with 100 bootstrap samples. First‐order indices represent the impact of each individual parameter change on model output, whereas the total‐order indices account for potential interactions among parameters. Sobol indices of each variable were depicted in a tornado chart.

For the parameters identified as most influential, we ran additional model iterations to characterize their individual impact on risk output, considering independent sampling one parameter at a time and a constant environment for the others. Specific R packages “mc2d” (Pouillot, 2022), “lhs” (Carnell, 2022), and “sensitivity” (Iooss et al., 2022) were used for the analysis.

### 2.3 Uncertainty assessment

Once the most influential input parameters had been identified, we quantitatively estimated how the uncertainty around their values could affect the accuracy of our model outputs, in both time and space. The general approach was to introduce variation in each influencing parameters according to their level of uncertainty, iterate model runs (500 iterations), and quantify statistical quantities among iterations of risk outputs. The methodology used to introduce variation (random sampling on probabilistic distributions) depended on whether influencing parameters were either published as a static point estimate, derived from an environment‐dependent function, or derived from a database (see details in Supplement Information S.2). In the absence of uncertainty indications in literature, noise of 10% (Yakob & Clements, 2013) was arbitrary introduced. For each destination *j* and for each week of the study period, we calculated P(IR>1), the proportion of iteration runs where $IR_{ij}>$1.

## 3 RESULTS

### 3.1 BTV infection at source (step 1)

**FIGURE 3:**
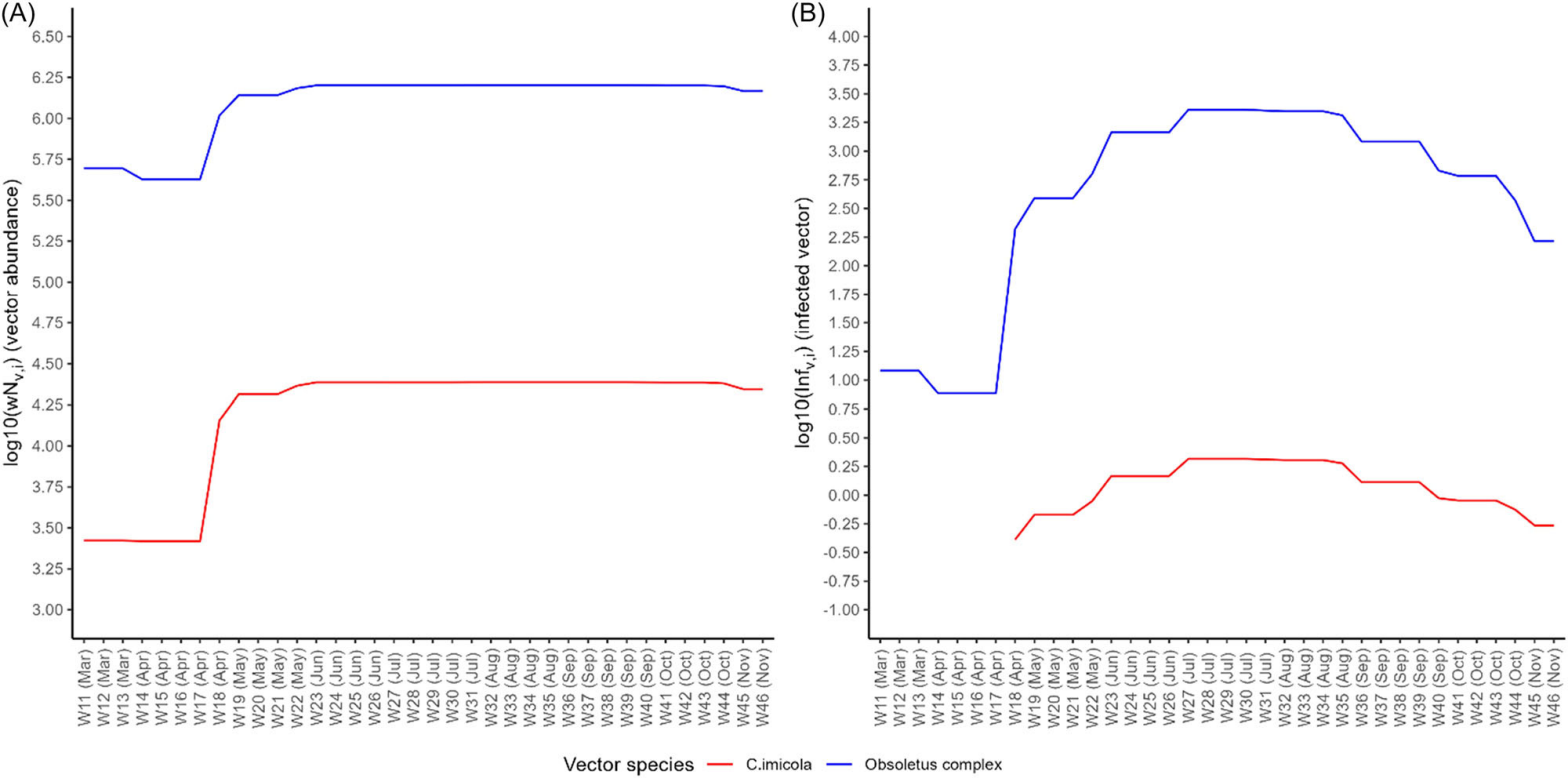
Weekly estimates of vector abundance (A) and infection (B) in the source area i (Sardinia), for the two‐vector species, in log10 scale. On the *x*‐axis, month name (in brackets) for each associated week number is indicative of the 2020 calendar.

Over the 3300 km^2^ source area, the maximal abundance of Obsoletus complex midges was 1.8 in log_10_ higher than for *C. imicola* midges (1.6 × 10^6^ vs. 2.4 × 10^4^ individuals) (Figure 3). Disease prevalence set at 0.06% in small ruminants and 0.66% in cattle led to a median number of 2.81 in log_10_ (equivalent to 654 individuals) infected Obsoletus complex midges per week and −0.04 in log_10_ (equivalent to 0.9 individuals) infected *C. imicola* midges per week, but with a significant variation range over the study period (Figure 3). Infection in the vector populations started to increase since mid of April (week 18) and peaked during summer (weeks 26–34—end of June to end of August) with a number of 2480 Obsoletus complex and 2 *C. imicola* infected vectors per week. The number of infected vectors started to decrease smoothly from September to mid‐November. Maximal infection rates, observed in July, were 1.6 × 10^−3^ and 8.2 × 10^−5^ for Obsoletus complex and *C. imicola*, respectively.

### 3.2 Probability of long‐distance wind dispersal (step 2)

**FIGURE 4:**
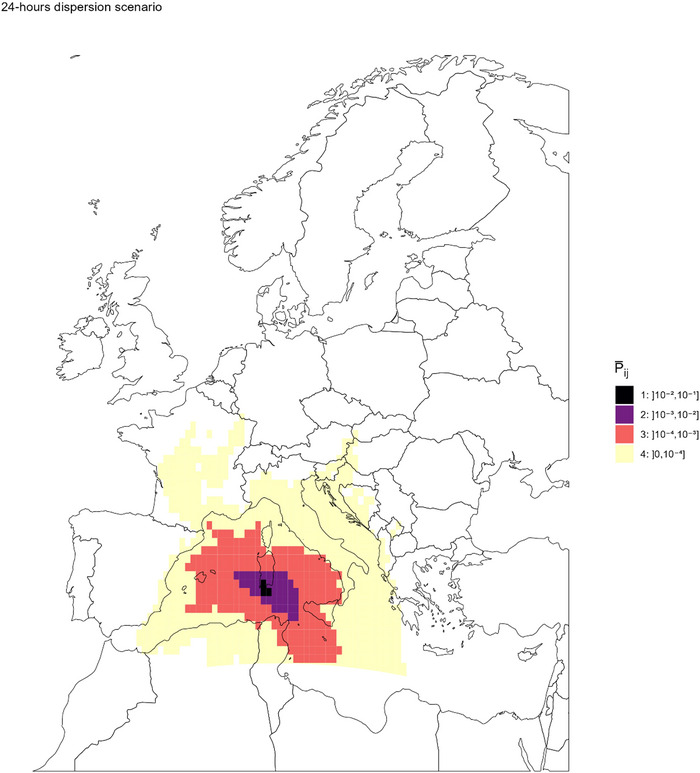
Spatial distribution of the overall mean probability of long‐distance wind dispersal from southwestern Sardinia to any destination in Europe (step2), considering 24‐h maximal flight duration and the survival conditions of Obsoletus complex (${\bar{P}}_{ij}$ equivalent to ${\bar{P}}_{2,\;ij}$ here). Here, overall mean probability ${\bar{P}}_{ij}$ is computed over the considered 36‐week study period.

Figure 4 depicts the four dispersion rings of the overall probability of LDWD ${\bar{P}}_{ij}$ from southwestern Sardinia. The dispersion ring (1) of highest probability ]10^−2^–10^−1^] was restricted to southern part of Sardinia. The dispersion ring (2) of probability between 10^−3^ and 10^−2^ covers half of Sardinia but never reached any other island. The dispersion ring (3) of probability between 10^−3^ and 10^−4^ covered western part of Sicily, the southwestern point of Italy, Malta, the Balearic Islands, and the northern and eastern borders of Tunisia, but not Corsica. The lowest probability dispersion ring (4) reached extreme destinations such as Paris in France, south‐eastern border of Spain, or eastern countries like Kosovo or Bosnia and Herzegovina.

When considering longer time of passive flight (Figure S3.3), the lowest probability dispersion ring (4) was much more broadly extended. After 48 h of dispersion, destinations as far north as southern Sweden (∼1800 km to Malmö) or the border between Ukraine and Belarus (∼2000 km) could be reached, but with very low probability. No western continental country was reached with a probability ${\bar{P}}_{ij}\;$> 10^−3^. Surprisingly, the overall probability of LDWD to Corsica was <10^−4^, whatever the scenario, despite being less than 260 km away from southwestern Sardinia.

**FIGURE 5:**
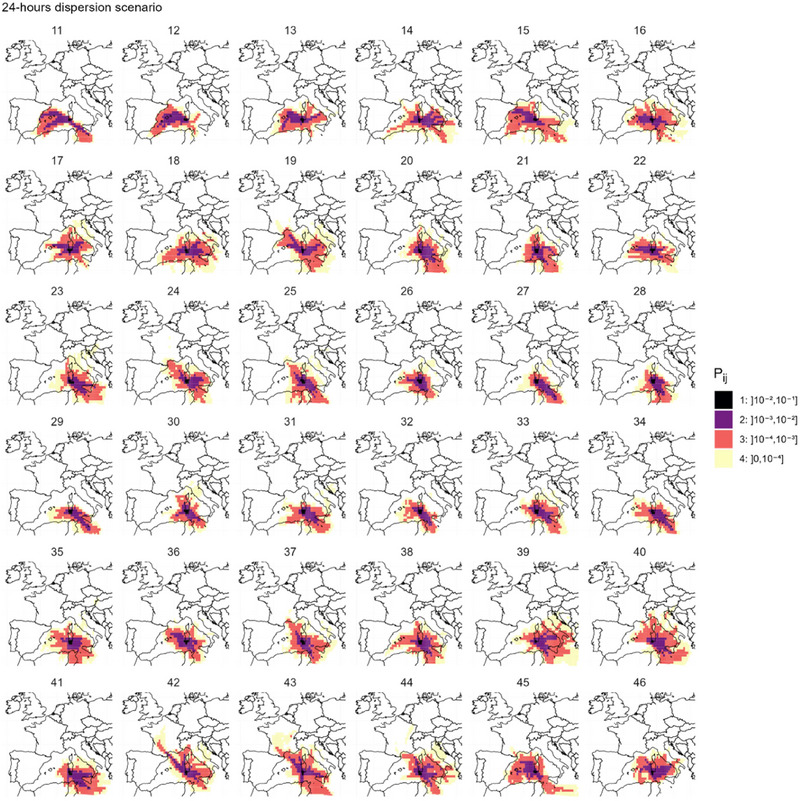
Spatial distribution of the mean probability of long‐distance wind dispersal from southwestern Sardinia, considering 24‐h maximal flight duration and the survival conditions of Obsoletus complex (${\bar{P}}_{ij}$ equivalent to ${\bar{P}}_{2,\;ij}$ here). Here, mean probability $P_{ij}$ is computed for each week of the study period, considering constant environmental conditions.

However, the spatial distribution of the overall expected probability LDWD ${\bar{P}}_{ij}$ from southwestern Sardinia hides marked temporal variations throughout the study period (Figure 5). Unlike in Figure 4, dispersion rings of $P_{ij}$ pointed to the western direction at the beginning of the study period, mainly from mid‐March to mid‐May (weeks 11–19) or by October (weeks 38 and 40). The dispersion ring (3) of $P_{ij}$ between 10^−3^ and 10^−4^ occasionally reached the Mediterranean coast of continental France (weeks 19, 24, and 37) and made a significant incursion over the southwestern part of the country (provinces of “Pyrénées Orientales,” “Aude,” and “Herault”) at weeks 42 and 43.

When considering 48 h of wind dispersal (Figure S3.4), the incursions over mainland France by the dispersion ring (3) were more frequent (observed 13 times over 36 weeks) and were more marked at weeks 24–25 (June) and weeks 42–43 (third week of October).

### 3.3 BTV transmission at destination (step 3)

**FIGURE 6:**
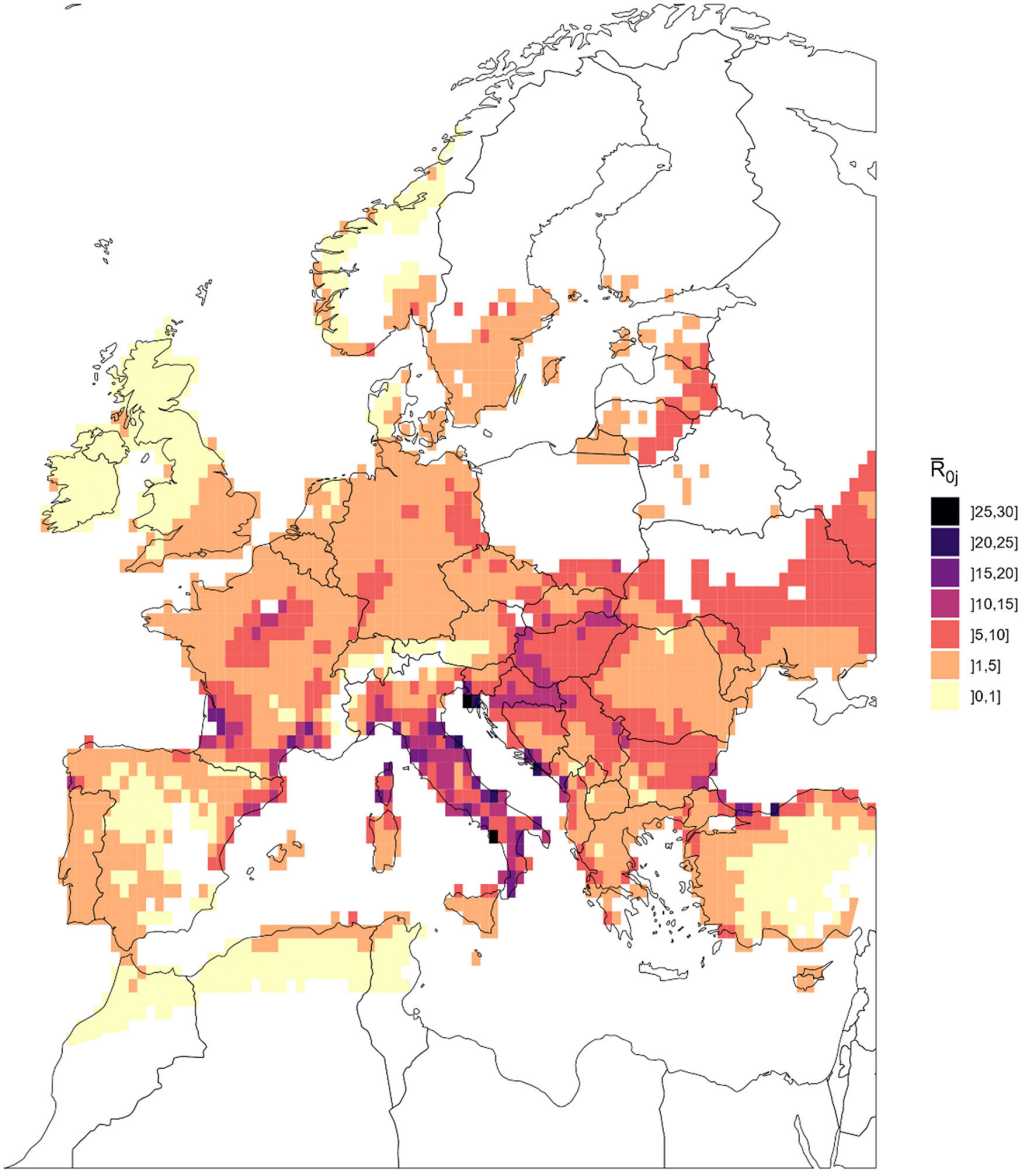
Spatial distribution of the overall transmissibility ${\bar{R_{0}}}_{j}\;$ of bluetongue virus (BTV) in Europe considering two‐host types (cattle and small ruminants) and two‐vector populations (*Culicoides imicola* and Obsoletus complex). Here, the values of ${\bar{R_{0}}}_{j}\;$ in each grid cell *j* are computed as the mean of $R_{0_{j}}$ over the study period and considering constant weekly environmental conditions.

Destinations with $R_{0_{j}}$ greater than 1 constitute areas where BTV is likely to be transmitted to local populations, considering two vector and host populations. Figure 6 shows the spatial distribution of ${\bar{R_{0}}}_{j}$, the mean of $R_{0_{j}}$ over the study period. In Italy, the whole peninsula was dotted with high‐risk areas of transmission, particularly on the coastal line and rarely on the northern region. In France, Corsica and three areas in the mainland country exhibited a high risk of transmission: southeastern Mediterranean coastal line, southwestern area, and northern‐central region surrounding the south of Paris. In Spain, the northeastern Mediterranean coast and the northwestern area were shown to be more at risk than the rest of the country. High values of ${\bar{R_{0}}}_{j}$ were also estimated on the eastern coastal line of the Adriatic Sea and the southwestern border of the Black Sea. Unsurprisingly, null or very low values were estimated in mountainous areas like in the Alps, the Pyrénées, central Spain, Scandinavian, and Balkan Mountains.

Maps of $R_{0_{j}}$ computed for each week of the study period (Figure S3.5) highlight seasonal variations of $R_{0_{j}}$ in line with variations of vector abundance. The disease transmission was unlikely ($R_{0_{j}}\;$< 1) in most of European destinations before week 17 (mid of April) and after week 44 (end of October). Although $R_{0_{j}}$ integrate the role of both vector species in the spread of BTV, it appears to be mostly driven by Obsoletus complex midges (Figure S3.6), probably due to its higher abundance and widespread distribution in comparison with that of *C. imicola* (Figure S3.1).

### 3.4 Introduction risk outputs ($IR_{ij}$)

**FIGURE 7:**
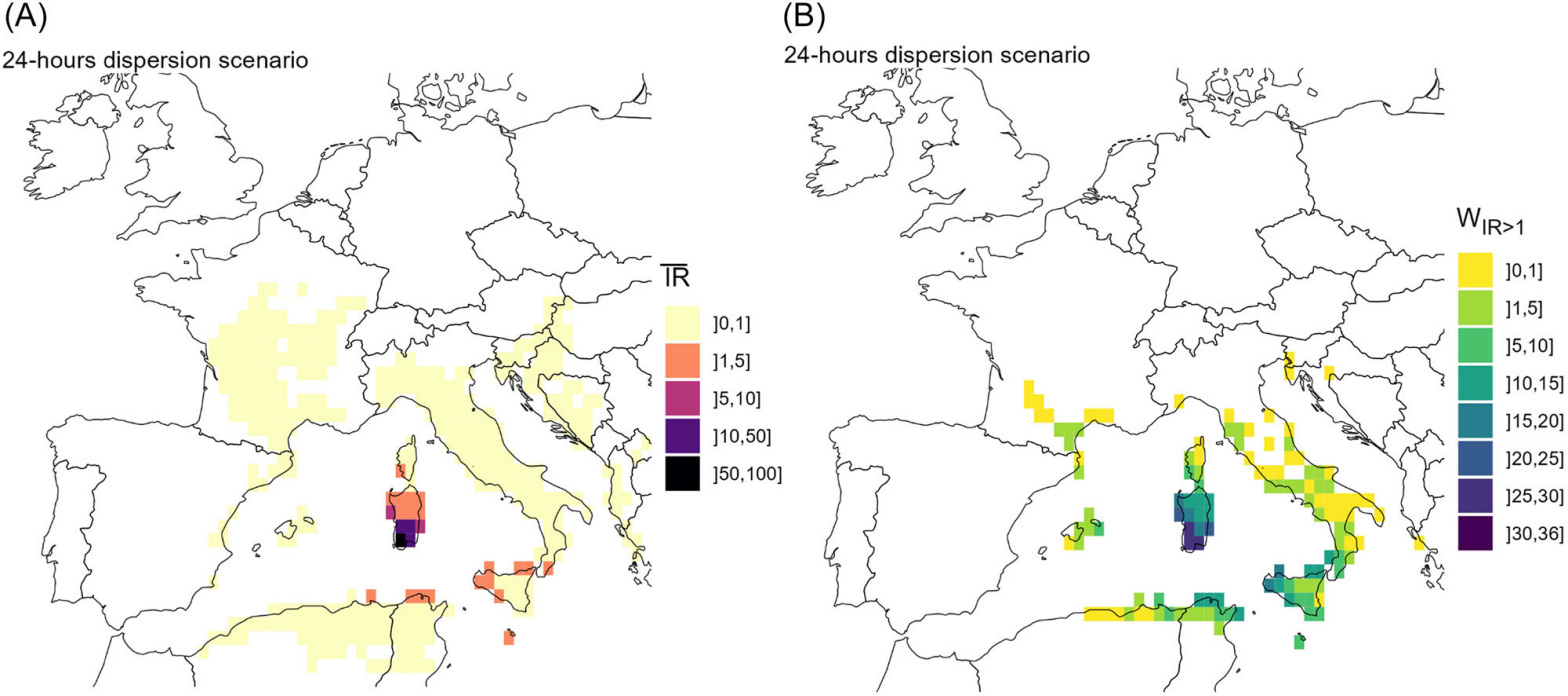
Spatial distribution of the overall risk of introduction from southwestern Sardinia to any destination in Europe, considering a 24‐h maximum flight duration of vectors. Here, the risk of introduction was expressed as (A) the overall risk of introduction ($\bar{IR}$) in Europe and (B) the number of weeks in which the mean risk of introduction IR is greater than 1 ($W_{IR>1}$). Here, the mean risk of introduction IR was computed considering constant weekly environmental conditions and a 24‐h maximum flight duration of vectors.

Figure 7A shows the spatial distribution of ${\bar{IR}}_{ij}$, the mean of $IR_{ij}$ over the study period. Unsurprisingly, the highest estimates of risk of BTV introduction, that is, ${\bar{IR}}_{ij}$ > 10, were restricted to the southern part of Sardinia, whereas dispersion to the northern part of the island was characterized by a ${\bar{IR}}_{ij}$ between 1 and 5. Estimates of ${\bar{IR}}_{ij}\;$> 1 were found in Sicily, Malta, the southwestern coast of Corsica, and on the southern tip of Italy. When considering longer dispersion duration (Figure S3.10), destination cells with ${\bar{IR}}_{ij}\;$> 1 extended westward to Minorca (Balearic archipelago), northward to the Mediterranean coast of continental France, and eastward to sporadic destinations within the central and southern parts of the Italian peninsula. No risk above 1 was reported for any European countries/regions above latitude N45°, whatever the scenario of dispersion. Interestingly, several areas along the northern coast of Tunisia and Algeria showed a risk of BTV introduction ${\bar{IR}}_{ij}\;$> 1.

Temporal dynamics of the risk of BTV introduction over the study period and for the different scenarios of maximal flight duration are shown in Figure S3.8 and S3.9. Considering a 24‐h maximal flight duration, some areas sporadically experienced a $IR_{ij}>$1 for a few weeks (Figure 7B). In mainland France, the Mediterranean coast and the southwestern region were exposed at week 24 (mid‐June), week 37 (mid‐September), and weeks 42–43 (end of October). In Spain, Minorca and, to a lesser extent, Mallorca showed a risk above 1 during 1–10 weeks within the June to September period. In contrast, continental Spain showed only limited risk of BTV incursion from Sardinia, with its extreme eastern point showing a $IR_{ij}>$1 only once or twice over the study period. The southern and central parts of the Italian peninsula were sporadically exposed to risk greater than 1 at least once during the study period, but the central part was exposed later in the year (in October, from weeks 39 to 44).

As dispersal duration increased (scenarios of 48‐h maximal flight duration—Figure S3.9), estimates of ${\bar{IR}}_{ij}$ between 1 and 5 extended to the whole south of Corsica, the Mediterranean border of mainland France, and sporadically on the Italian peninsula.

Considering *C. imicola* alone as a competent vector to transmit the disease, overall risk of further incursion ${\bar{IR}}_{ij}$ remains below 1, whatever the scenario of dispersal duration considered (Figure S3.7).

### 3.5 Sensitivity analysis

**FIGURE 8:**
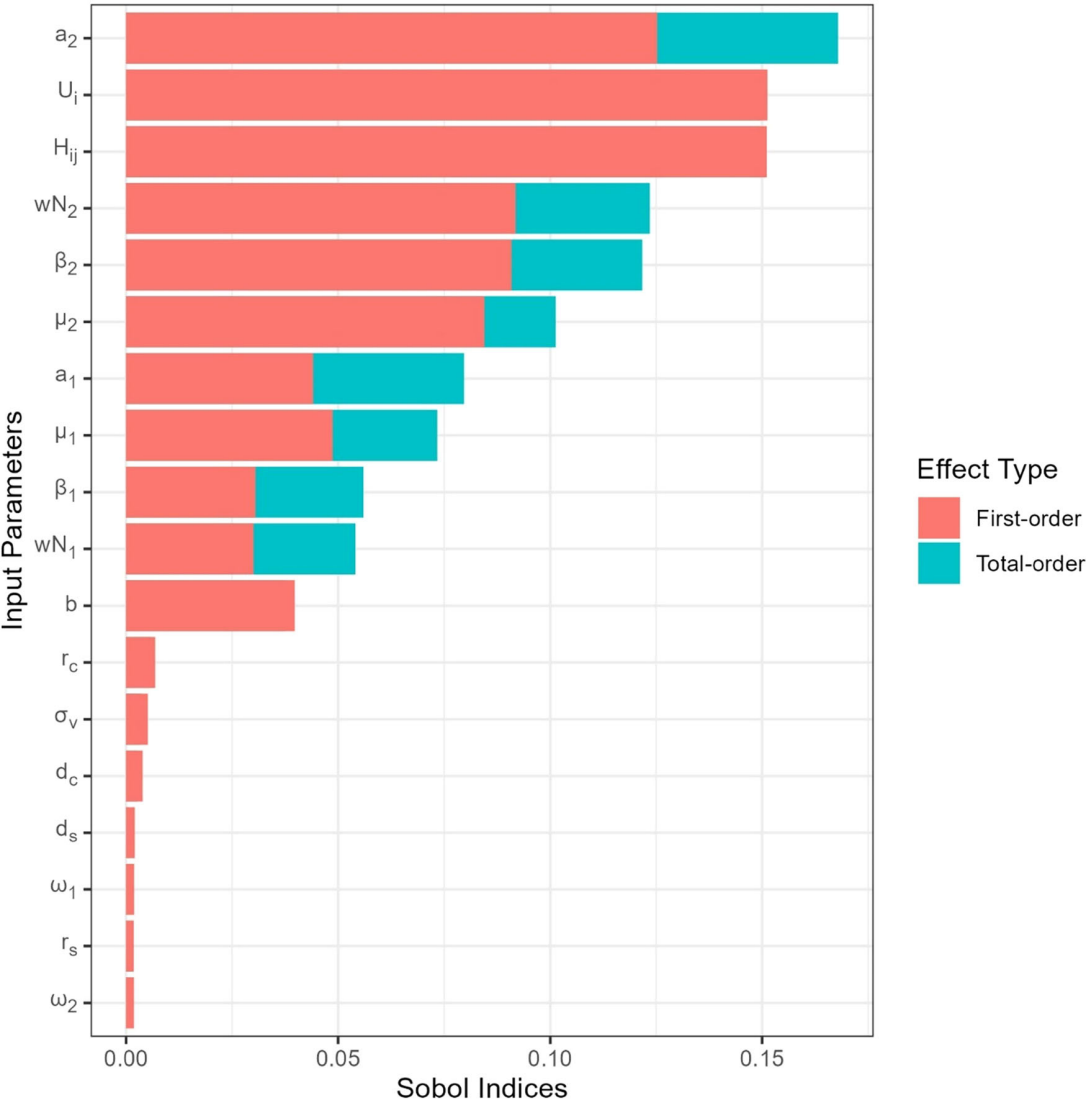
Results of sensitivity analysis ranking most influential parameters on model variability.

As expected, uncertainties in the probability to be uplifted in the windstream ($U_{i})\;$ and the HYSPLIT probability to be airborne to a new destination ($H_{ij})$ have the strongest impact on the model outputs, both independently (first effect) and in interaction with the other variables (total effect) (Figure 8). Uncertainties in parameters specific to the dynamics of Obsoletus complex midges and their role in disease transmission, such as biting rate ($a_{2})$, abundance ($wN_{2})$, transmission rate from host to vector ($\beta_{2}),\;$ and mortality rate ($\mu_{2})$, have also a significant impact on the model outputs, mostly when considering the interactions with other parameters. Their individual impacts on the risk outputs (function shapes and directions) are presented in Supplement Information S2.2.

Uncertainty on the *C. imicola*‐specific parameters, mostly the biting and mortality rates ($a_{1},\;\mu_{1})$, as well as the transmission rate from vector to host (b), showed a limited impact on the model. The vector preference for a host species $\left(\sigma_{v}\right)$, the host‐specific parameters (mortality and recovery rates of both host species), and the virogenesis rates of both species of vectors ($\omega_{1}$, $\omega_{2}$) do not have any impact on the model variance.

### 3.6 Uncertainty assessment around risk

**FIGURE 9:**
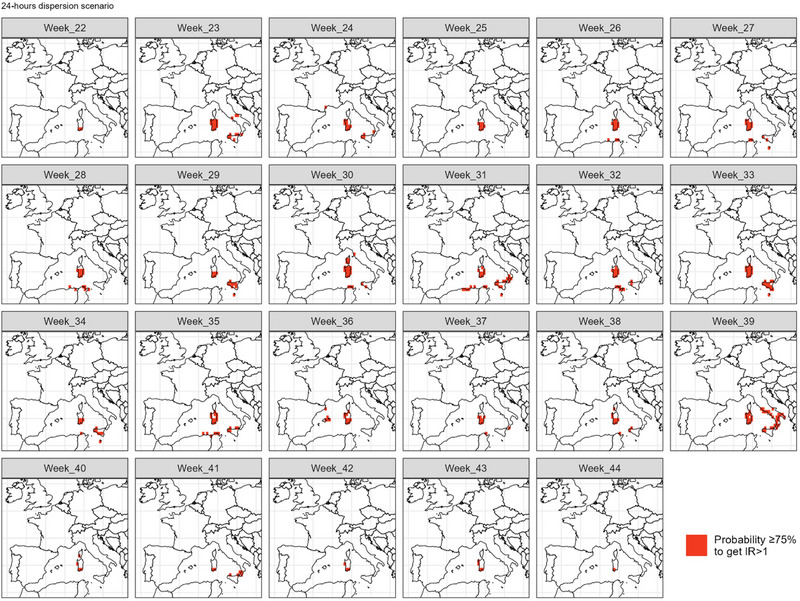
Areas in Europe showing a high estimated mean risk of introduction from southwestern Sardinia with high level of certainty. Uncertainty is measured as the probability *P_IR>_*_1_ for which the estimate of IR is greater than 1, considering uncertainty around key parameters. We considered that values of *P_IR>_*_1_ ≥0.75 show a high level of certainty. Here, estimates of IR were computed for each week of the study period and considering constant weekly environmental conditions and a 24‐h maximum flight duration of vectors.

Figure 9 highlights the destination cells that have a probability greater or equal to 75% of being at risk of introduction P(IR>1)≥0.75, when accounting for uncertainty in influential parameters (full estimates in Figure S3.11). In this analysis, no destination could be identified at risk (IR>1), with at least 75% confidence, before week 22 (end May/beginning June) and after week 44 (end October), which is 5 weeks less than for the baseline period when uncertainty is not considered (from week 19—beginning May—to week 46—mid‐November). Sicily and the southern tip of the Italian peninsula remained the main risk areas, although the central and northern parts of the peninsula were exposed sporadically for only 1 or 2 weeks. Interestingly, Northern coast of Tyrrhenian Sea and Corsica were simultaneously at risk on week 30 (end of July). In addition, southwest of Corsica is also exposed later in the summer.

Week 24 (mid‐June) was the only week during which mainland France was exposed to a risk greater than 1 with a high confidence level. For Minorca and the southeastern Mediterranean border of mainland Spain, the riskiest week was week 36 (September).

## 4 DISCUSSION

In this study, we quantified the risk of BTV‐3 introduction as a mean daily rate of new infections expected at destination per week, proportionally to the number of vectors that were infected in the southwest of Sardinia, successfully brought by the wind and did infect local populations at destination. Variations in time and space of this risk estimate were used to predict areas and periods at risk throughout Europe.

First, our results confirmed a high risk of northward expansion of BTV within Sardinia Island, which was historically evidenced for other BTV serotypes (Calistri et al., 2004) but surprisingly not for BTV‐3 since 2018. One hypothesis could be that the Obsoletus complex midges have a reduced competence (lower transmission rate from host to vector) against this viral serotype than the one used in our model. No specific vector competence study was done specifically for Obsoletus complex for this strain, and recent entomological trappings in the North of the source area failed to identify Obsoletus complex midges infected by BTV‐3, contrary to *C. imicola* (Quaglia et al., 2023). Under the hypothesis that Obsoletus complex would not be competent to replicate the Sardinian BTV‐3 strain, the unique *C. imicola* population seems not sufficient to constitute a risk of BTV spread by the wind (Supplement Information S3.7). However, the recent BTV‐3 outbreak in the Netherlands (WBVR, 2023) supports the fact that Obsoletus complex midges might be able to transmit BTV‐3 virus strains, at least the one circulating in the Netherlands. Further studies on the vector competence of the Obsoletus complex with respect to both BTV‐3 strains would be of particular interest to fine‐tune our model.

Out of Sardinia, the riskiest destinations for further dispersion are Sicily, Malta, and the southern tip of Italy, due to a major windstream blowing in that direction. This result was observed at both temporal scales (weekly and annually) and remained robust despite uncertainty of the input parameters. This southeastern spread is in line with history of BTV‐2 which emerged in August 2000 in Sardinia and was successively introduced in Sicily and in the Calabria region of the Italian peninsula (Calistri et al., 2004). Corsica and Minorca were also shown to be at risk of introduction but more sporadically during specific weeks of the study period. Here again, it appears consistent with historical cases of BTV‐4, which was first detected in Sardinia (August 2003) and subsequently reported on Corsica and Minorca (October 2003) (Gómez‐Tejedor, 2004). Regarding Balearic archipelago, our results suggest a lower risk for Mallorca than for Minorca, whereas historical cases of BTV‐2 were first reported on Mallorca and subsequently on Minorca in 2000 (Calistri et al., 2004; Diego et al., 2014). However, it should be noted that BTV‐2 was concomitantly circulating in North Africa in 2000, suggesting that these countries may have been the original or additional sources of BTV‐2 introduction to the Balearics (Burgin et al., 2013). Additionally, our results support a direct introduction from Sardinia to Tuscany region in Italy as was suggested by Purse et al. (2008) for BTV‐2. The risk of introduction into mainland France and Spain was low, sporadic, and uncertain considering a 24‐h maximal flight duration but increases when the maximal flight duration was considered longer. Although these regions have never reported cases of BTV‐3, they should be considered surveillance hotspots. The first cases of BTV‐3 in the Netherlands were diagnosed in the region of Utrecht (WBVR, 2023), in early September 2023. As this location was not identified by our model as a high‐risk destination for the introduction of BTV‐3 by long‐distance dispersal of vectors from Sardinia, whatever the scenario of maximal flight duration, it can be suggested to investigate other routes of introduction such as trade of live animal and germplasm or aircraft movements. Finally, despite the lack of a true validation process, our framework was able to predict variations of risk in time and space, consistent with previous BTV incursion to guide surveillance at large scale.

Although our model shows consistent results with historical empirical evidences, we acknowledge several limitations at each step of our model framework.

First, vector abundance data were estimated from VectorNet database (Balenghien et al., 2020) which has the advantages to be open access and gathering light trappings data from a huge entomologist surveillance network covering 28 countries of continental Europe and surrounding regions, from 2015 to 2018 (Balenghien et al., 2020). We assumed that the VectorNet metric (maximum number of midges per trap per day) could be equivalent to an average “aggressive” density (Balenghien et al., 2020) per km^2^ (or a mean density per farm in a pixel of 1 km^2^), with a constant renewal of the vector population. Knowing that trappings data do not represent the absolute *Culicoides* abundance (Meiswinkel & Elbers, 2016), and the attraction range of a light trap is lower than 1 km^2^ (Kirkeby et al., 2013) and varies between species (Viennet et al., 2013), vector abundance estimates used in the model might be underestimated. Moreover, the maximal abundance retrieved for *C. obsoletus/scoticus* in the source area was about 63 times higher than for *C. imicola*, whereas Italian field trappings normally collect higher *C. imicola* than *C. obsoletus* in this low‐altitude area of Sardinia (Foxi & Delrio, 2010; Foxi et al., 2016). This discrepancy may suggest that the VectorNet's algorithm, despite its overall good performance, tends to overestimate abundance for vectors that are ubiquitous and widely distributed such as Obsoletus complex, and for which, the lack of data reporting an actual absence compromises the predictive performance of the model. Weekly abundances were calculated applying a presence probability function *w* to the maximal vector abundance, and this function was applied for both vector species at European scale, whereas it was initially established for *C. imicola* in Italy (Conte et al., 2003). However, the large activity period from spring to late autumn was consistent with the one usually reported. But we could not reproduce, specifically for Obsoletus complex in southern areas, the reduced peak of activity in summer when the conditions are dry and hot (Versteirt et al., 2017). For all these reasons, our estimates of weekly abundance might be biased, particularly for Obsoletus complex, and should be improved by continuous trapping collections and better understanding around its ecology.

Second, disease prevalence in host in source i (0.06% in small ruminants and 0.66% in cattle) was considered constant over the study period in absence of official reports of BTV‐3 cases per month. Although these estimates can be classified as “low” and “moderate,” respectively, according to the MintRisk scoring for vector‐borne disease (Vos et al., 2021), they might overestimate the infection pressure when the vector abundance is low, particularly in the beginning of the period. Despite this conservative assumption, the final risk outputs remained low.

Third, a higher infection rate in Sardinia was calculated for Obsoletus complex than for *C. imicola* (maximum rate of 0.16% and 0.008%, respectively). Although this is in line with more favorable transmission parameters suggested for this first species (Foxi et al., 2016), comparison with field estimations of infection rate derived from vector capture suggests that our estimates appear lower than those reported during the BTV‐1/BTV‐4 epidemy in Sardinia (Goffredo et al., 2015) (3.6% for Obsoletus complex and 1.7% for *C. imicola* specifically) and little higher than estimates for BTV‐9 from the Italian peninsula for Obsoletus complex (0.005%) (Braks et al., 2017; Savini et al., 2005). Vector infection rates reported in literature are heterogeneous due to diverse calculation methodologies (Braks et al., 2017) (pool vs. individuals) or disease context (epidemic vs. endemic situation) and differ a lot between virus serotypes. Specific BTV‐3 laboratory studies assessing infection, dissemination, and transmission rates would be valuable for our model parametrization.

One of the greatest strengths in our methodology is to forecast vector airborne dispersion from atmospheric simulations (step 2) based on real meteorological data and considering their variations at a fine spatial (grid cells of 2500 km^2^) and temporal (weekly) scales. This enables us to precisely identify wind pathways that would be overlooked at higher resolution or timescales. Indeed, HYSPLIT simulations predicted some sporadic wind incursions to the northwest direction toward continental France in June and October, despite a global wind pattern pointing in the southwestern direction. These episodic events may be linked to the northward Sirocco wind flow coming from Sahara Desert, known to result in hot and dusty climatic conditions in European countries such as Spain (Sousa et al., 2019), Portugal, and France. Global climate models have identified the Mediterranean area as a hot spot for climate change with winter rain decline and increasing wind and temperature anomalies (Tuel & Eltahir, 2020). To integrate these future meteorological changes into our model and to guarantee reliable estimations, regular updates of HYSPLIT simulations will need to be performed. Importantly, the sensitivity analysis confirmed that the probability of wind dispersion is one of the most influencing parameters on the risk output (Figure 8). This result highlights the importance of the methodological filters applied to raw simulations to ensure that final trajectories best mimic the flight of a living and active midge. First, the impact of the maximal flight duration was assessed through two scenarios considering a maximal flight duration lasting for 24 or 48 h, unlike most of the previous studies that defined only one duration, between 12 and 72 h (Alba et al., 2004; Burgin et al., 2013; Eagles et al., 2012, 2013; García‐Lastra et al., 2012; Jacquet et al., 2016; Klausner et al., 2018; McGrath et al., 2018; Rajko‐Nenow et al., 2020). The 12‐h flight duration limit has been supported by some unpublished data reporting that *Culicoides* midges tethered in a blowing tunnel were able to fly for 10 consecutive hours (Burgin et al., 2013). For most of the other studies, no clear justification of the threshold was provided. In absence of robust experimental evidence, we originally judged plausible the two scenarios (and even a third one considering 72 h of dispersion but not presented here) in accordance with vector lifespan, ranging between 14 days and 6 weeks. These long durations of flight were also the opportunity to test extreme situations, the most conservative in terms of risk. However, when considering results from 48‐h maximal flight duration scenario (Supplement Information S3), the insects could be air‐borne from Sardinia over an extensive and maximal distance up to 2000 km at a probability lower than 10^−4^. Although this probability was very low, such a long distance did not appear consistent with past BTV introductions in Europe. Moreover, no marked difference was observed between 48‐ and 72‐h maximal flight duration scenarios, and the risk of introduction, most of the time, did not exceed the Mediterranean basin (results of the 72‐h scenario not presented in this article). In this context, we believe that the 24‐h scenario could be the most realistic for future studies as the predictions were consistent with historical BTV incursions from Sardinia.

Another filtering condition that was used to infer the probability of air‐borne dispersion was the temperature of the air‐mass transporting the insect. A cut‐off was set at 10°C in accordance with the minimal isotherm for *Culicoides* established between 9.7 and 12.5°C (Conte et al., 2003; Sellers & Mellor, 1993). Such strict limit could be challenged as each *Culicoides* species might tolerate different conditions (Murray, 1995; Venter et al., 2019). To our knowledge, no temperature threshold was previously considered when assessing the risk of vector dispersal due to wind. Some other authors used a survival function depending on temperatures (ADS model) (Aguilar‐Vega et al., 2019; Fernández‐Carrión et al., 2018) or limited simulations height to 1000 m (Eagles et al., 2013). These temperature thresholds, together with the PBL, are important elements to account for the topography. As expected, our results estimated a very low or null dispersion probability in mountainous areas. This may partly explain the very low dispersal probability observed in Corsica despite its proximity to Sardinia (Figure 4).

Regarding the HYSPLIT simulations, we favored the use of the trajectory mode rather than the concentration mode (prediction of a particle density deposited at destination knowing a fix number released at the source), unlike other applications that used it alone or in combination of both (Aguilar‐Vega et al., 2019; Durr et al., 2017; Eagles et al., 2013; McGrath et al., 2018). We considered that flying vectors could not behave like gases or inert particles, characterized by their physical parameters (such as density or drag coefficients) that determine their likelihood to be deposited but could maintain themselves in the air, even during passive flight with minimal energy. In addition, the number of particles released at the source, which is fixed in the concentration mode, was found to be highly variable in literature (2500–100,000) (Eagles et al., 2013; McGrath et al., 2018) and not supported by empirical data. Finally, our approach aimed to limit complexity and sources of uncertainty according to the principle of parsimony.

The probability for the *Culicoides* spp. to be uplifted in a windstream was identified as a critical but highly uncertain parameter (Figure 8). In absence of any experimental study in this field from our knowledge, we took a constant and conservative value of 25% of the weekly infected vectors that could enter an ascendant windstream up to 50 m a.g.l. (minimal altitude for HYSPLIT to efficiently simulate atmospheric trajectories) and be subsequently windborne transported. However, this estimate is much higher than the range 10^−5^–10^−3^ per day (7.00.10^−5^–6.97.10^−3^ per week) estimated by Hall et al. (2022), irrespective of the insect type among midges, mosquitoes, or heavy flier like tabanids. In contrast to this study, we restricted the simulation start time window to dusk and dawn, which are more suitable times for take‐off with ascendant airstream than turbulent daytime (Reynolds et al., 2006). *Culicoides* midges, due to its very small size (1–3 mm), are more likely to be windborne transported and found at high altitude (2 km) (Chapman et al., 2004; Reynolds et al., 2006; Sanders et al., 2011) than mosquitoes or big flies. We judged then our estimate plausible but uncertain and included a large range of variation in the uncertainty assessment. Despite this conservative estimate of $U_{i}$, the predicted at‐risk locations remain limited.

When calculating the BTV basic reproduction number in such large geographical scale as Europe (step 3), discrepancies of spatial distributions and abundances between vector species need to be considered. In this case, the two‐host, two‐vector $R_{0}$ formula used in our model (Turner et al., 2013) is believed to provide a better estimate of disease transmission potential than the two‐host one‐vector formula (Gubbins et al., 2008). However, in our model, *C. imicola* contributed very little to final $R_{0}$, which might explain lower $\;R_{0}$ values in southern European regions than usually calculated (Guis et al., 2012). In Spain, our results showed high risk of BTV transmission not only at the northern border, similarly illustrated by Aguilar‐Vega et al. (2020), but also at the northeastern Mediterranean border close to France. In the Netherlands, our $R_{0}$ values were lower and more homogeneous than illustrated by Hartemink et al. (2009) for Obsoletus complex, probably because we used a lower resolution grid. In France, surprisingly, areas of high BTV transmission were evidenced around Paris and Bordeaux cities where past BTV outbreaks were rare. The areas were characterized by low densities of ruminant hosts but high densities of Obsoletus complex midges leading to high value of vector/host ratios. It is important to note that this $R_{0}$ approach considers the host population as fully susceptible, which might not be the case in countries where a vaccination program is implemented or previous outbreaks have occurred. Another improvement of the $R_{0}$ could be to consider additional vector species in the formula as long as they are shown competent to transmit BTV, such as *Culicoides newsteadi* (Foxi et al., 2019; Kundlacz et al., 2019).

Seasonal variations of $R_{0}$ estimates were observed during the study period (Supplement Information S3), principally due to the weekly vector abundance as discussed previously, but also due to the vector transmission parameters (biting rate, transmission rate from host to vector, mortality rates…), which are all functions of ambient temperature (Haider et al., 2019; Mullens et al., 2004; Turner et al., 2013; White et al., 2017), considering the poikilothermic nature of the arthropod. These functions were mostly retrieved from studies involving the American species *Culicoides sonorensis* and not Obsoletus complex, due to the difficulty of the latter to be bred in laboratory conditions. Nevertheless, functions used in the model led to higher biting rate, higher vector‐to‐host transmission, and lower mortality rate for Obsoletus complex midges as compared to *C. imicola*, in line with literature reporting a better vectorial capacity, higher Minimum Infection Rate (Foxi et al., 2016), increased longevity (Meiswinkel et al., 2008), and lower isotherm (Versteirt et al., 2017). Further experiments investigating specifically Obsoletus complex midges would be beneficial to reduce uncertainty around the function's parameters.

Globally, our model computes a high number of parameters that can have a significant impact on the final risk outputs (Figure 8, Supplement Information S2.2). Among the most important is the probability to be uplifted in the air‐mass $U_{i}$ and the parameters specific to the Obsoletus complex (abundance, competence for the BTV‐3 strain, and activity rate), which were probably overestimated with respect to the assumptions made, the literature available, and the data used in the model. Even under these conservative conditions, our results suggest that the risk of BTV‐3 introduction outside Sardinia remains limited to the southern Mediterranean Basin. The destinations identified at‐risk remain consistent when accounting for uncertainty in most influential parameters, but the number of weeks a destination was exposed to a risk >1 was reduced, as was the total risk period between the end of May and the end of October (Figure 9).

As mentioned above, most of the parameters of our model were not serotype specific. Therefore, the location of the source determines the specificity of the model application, and here the southwestern part of Sardinia is the main factor that makes this assessment specific to BTV‐3. On the other hand, this model constitutes a generic framework that can be easily reused for other BTV serotypes present in different locations, provided that HYSPLIT simulations are available from that source. Adaptation of the model to other *Culicoides*‐borne diseases, such as Epizootic Hemorrhagic Disease Virus, can also be envisioned, provided that the transmission pattern and competent vectors can be considered similar to BTV.

## 5 CONCLUSION

In this study, we assessed the risk of BTV introduction by long‐distance dispersal of infected vectors considering meteorological data and vector biological limits during flight. The at‐risk destinations identified by the model applied for BTV‐3 are consistent with historical data for other serotypes and provide valuable information that may facilitate preparedness and target surveillance. Further experimental investigations on *Culicoides* flight conditions and Obsoletus complex‐specific parameters are needed to improve the robustness of our predictions and continue efforts in preventing arbovirus emergence.

## Supporting information

SUPPORTING INFORMATION

SUPPORTING INFORMATION

SUPPORTING INFORMATION

## Data Availability

Raw data regarding HYSPLIT atmospheric simulations are openly available in data INRAE repository within the “Experimental—Observation—Simulation” dataverse, with the following reference link: https://doi.org/10.57745/IS51BX. Other data can be available upon request to the corresponding author.
