# Supplementary material for: Quantitative risk assessment for the introduction of bluetongue virus into mainland Europe by long‐distance wind dispersal of Culicoides spp.: A case study from Sardinia
Source: Risk Anal. 2024 Jul 2;45(1):108–27. doi: 10.1111/risa.14345 (PMC11735344; doi:10.1111/risa.14345)
Supplement: Supplementary file 1 — SUPPORTING INFORMATION [file RISA-45-108-s001.docx]

**Quantitative risk assessment for the introduction of Bluetongue virus into mainland Europe by long-distance wind dispersal of Culicoides spp. : A case study from Sardinia.**

Supplementary material S1

# S1.1 Calculation of the number of infected vectors in source area $i$

We aimed at describing the dynamic of infection in the vector populations in source area $i$. We considered 2 vectors populations of type$v$, respectively 1 for *C. imicola* and 2 *C.* Obsoletus complex (*Obsoletus/Scoticus*), and 2 hosts populations of type $h$ , respectively C for Cattle and S for Small ruminants (assuming same parameters and pattern for sheep and goats). The proportion of infected hosts among the total population of hosts in denoted $prev_{h}$.

In $i$, the numbers of susceptible, latent and infectious vectors - denoted by $S_{v}$ , $L_{v}$ and $I_{v}$ respectively and the total population of vectors is denoted $N_{v}$, with $v$ as the type of vectors.

The evolution with time of the numbers of individuals in each compartment is described as the derivatives below (Turner, Bowers, & Baylis, 2013):

$$\frac{dS_{v}}{dt}=\rho_{v}N_{v}-{{\lambda_{V}}_{v}S}_{v}-\mu_{v}S_{v}$$

$$\frac{dI_{v}}{dt}=\omega_{v}L_{v}-\mu_{v}I_{v}$$

$$\frac{dL_{v}}{dt}={\lambda_{V}}_{v}S_{v}-\left( \omega_{v}+\mu_{v} \right)L_{v}$$

With:

$\rho_{v}$= replacement rate of vector type $v$

$\mu_{v}$ = mortality rate of vector type $v$

$\omega_{v}$ = virogenesis rate, *i.e*. rate at which latent vector of type $v$become infectious (1/Extrinsic Incubation Period in days)

$\lambda_{V_{v}}=\sum_{h\left\{ C,S \right\}} \beta_{v}a_{v}{(\phi}_{hv}*prev_{h})$ named “force of infection”(Turner et al., 2013)

$prev_{h}$ = proportion of infectious host type $h$ (= number of infectious hosts $h$ /total host pop of type $h$) = disease prevalence in host of type $h$

$\phi_{hv}$= proportion of vectors of type $v$attracted by hosts type $h$

$a_{v}$ = biting rate of vectors of type $v$,

$\beta_{v}$ = probability of effective virus transmission from a host to a vector of type $v$ given an infection in vector of type $v$ (effective contact).

As in area $i$, the disease status is considered at the endemic equilibrium:

$$\frac{dI_{v}}{dt}=\omega_{v}L_{v}-\mu_{v}I_{v}=0$$

$$\begin{aligned} I_{v}=\frac{\omega_{v}}{\mu_{v}}L_{v}\#( SEQ ( \backslash* ARABIC 1 ) \end{aligned}$$

And

$$\begin{aligned} \frac{dL_{v}}{dt}={\lambda_{V}}_{v}S_{v}-\left( \omega_{v}+\mu_{v} \right)L_{v}=0 \end{aligned}$$

$\begin{aligned} L_{v}=\frac{{\lambda_{V}}_{v}}{\left( \omega_{v}+\mu_{v} \right)}S_{v}\#\left( 2 \right) \end{aligned}$

In $i$, we were interested in calculating the cohort of vectors that are infected, *i.e*. the sum of the latent and the infectious vectors : $I_{v}+L_{v}$. Combining equations (1) and (2), we obtain:

$$\begin{aligned} I_{v}+L_{v}=\frac{\omega_{v}}{\mu_{v}}\frac{{\lambda_{V}}_{v}}{\left( \omega_{v}+\mu_{v} \right)}S_{v}+\frac{{\lambda_{V}}_{v}}{\left( \omega_{v}+\mu_{v} \right)}S_{v}\#\left( 3 \right) \end{aligned}$$

Incorporating the previous expression of the vector force of infection, it leads to:

$$I_{v}+L_{v}=\frac{S_{v}(1+\frac{\omega_{v}}{\mu_{v}})}{\left( \omega_{v}+\mu_{v} \right)}\sum_{h=\left\{ C,S \right\}} \beta_{v}a_{v}{(\phi}_{vh}prev_{h})$$

Then the number of infected vectors $Inf_{v}$ can be expressed as:

$${Inf_{v}= I}_{v}+L_{v}=S_{v} \frac{1}{\mu_{v}}\sum_{h=\left\{ C,S \right\}} \beta_{v}a_{v}{(\phi}_{vh}prev_{h})$$

Assuming that the number of susceptible vectors can be approximated by the vector abundance in $i$:

$$Inf_{v}=N_{v} \frac{1}{\mu_{v}}\sum_{h=\left\{ C,S \right\}} \beta_{v}a_{v}{(\phi}_{vh}prev_{h})$$

For *Culicoides imicola* in area $i$:

$$\begin{aligned} {Inf}_{i,1}=N_{i,1}\frac{1}{\mu_{1}}\left( \beta_{C1}a_{1}\phi_{C1}prev_{i,C}+\beta_{S1}a_{1}\left( 1- \phi_{C1} \right)prev_{i,S} \right)\# \end{aligned}$$

For *Culicoides Obsoletus/scoticus* in area $i$:

$$\begin{aligned} {Inf}_{i,2}=N_{i,2}\frac{1}{\mu_{2}}\left( \beta_{C2}a_{2}\phi_{C2}prev_{i,C}+\beta_{S2}a_{2}\left( 1- \phi_{C2} \right)prev_{i,S} \right)\# \end{aligned}$$

The respective parameters used in the model are described in the paper.

# S1.2 Estimation of the weekly midge abundance

Considering the expected vector abundance of species$v$ in a given week *t*, $N_{v, t}$, such as

$$N_{v, t}\sim w.N_{v}$$

where$N_{v}$ is the maximal abundance reported within a given year and extracted from the VectorNet data; and $w$ is a probability function of midge presence and depending on environmental conditions. In line with results from Conte et al.(Conte et al., 2003), we considered *w* being driven by the minimal temperature, the minimal relative humidity and the average elevation above sea level recorded in each grid cell and for each week of the study period, such as:

$$w=\frac{e^{z}}{1+ e^{z}}$$

and

$z = \beta_{0}+ \sum_{k=1}^{K} \beta_{k}x_{k}$

where $x_{k}$ are the average values of the considered environmental condition in each grid cell and each week of the study period. The value and definition of $\beta_{k}$ and $x_{k}$ are shown in Table S1.2.1

Table S1.2.1. Details of the coefficient, and standard deviation, for all k variables found influencing the probability of midge presence *w* (Conte et al., 2003).

| *k* | Variable ($x_{k}$) | Coefficient ($\beta_{k}$) | Standard deviation |
| --- | --- | --- | --- |
| 1 | Minimal temperature (°C) | 1.166 | 0.110 |
| 2 | Average elevation above sea level (m) | -0.003 | 0.001 |
| 3 | Minimal relative humidity (%) | 0.498 | 0.062 |
| 0 | constant | -38.964 | 4.148 |

# S1.3 Databases computation

- *Host population*: cattle and small ruminant density were retrieved from the Livestock Grid GLW4 (Gilbert et al., 2018) (initial resolution of ~10 km) and the number of heads for each species was summed over the study grid. Sheep and goat heads were summed to compute small ruminant data. A minimal threshold of 1 animal per km² (2,500 animals per grid cell of 50 km x 50 km) was considered to compute the model.
- *Vector population*: Maximal abundance of *C. imicola* and Obsoletus complex in Europe were retrieved from VectorNet Data Series3(Balenghien et al., 2020) (initial resolution of ~1 km). Crude downloaded values were exponentiated and then summed over the study grid. A minimal threshold of 1 vector per km² was considered to compute the model. The standard deviation provided were squared to obtain variance, then summed over the final grid and finally square-rooted back to obtain standard deviation estimates at final grid.
- *Meterological data*: monthly average temperature, monthly minimal temperature and monthly average relative humidity were retrieved from CHELSA(Karger, Schmatz, Dettling, & Zimmermann, 2020) database for month No. 3 to month No. 11 and years from 2017 to 2019 for temperature (average and min) and years 2016-2018 for relative humidity (only monthly data available). The average over the 3 years period were used as the monthly input values. Weekly estimates were inferred from monthly inputs based on a 2020 calendar. For weeks straddling 2 months, the monthly estimate was assigned to each day of the week and the final monthly value was then calculated as the average of the daily values.
- *Altitude data* : elevation above sea level was retrieved from the European Environment Agency ([World digital elevation model (ETOPO5) — European Environment Agency](https://www.eea.europa.eu/data-and-maps/data/world-digital-elevation-model-etopo5) [(europa.eu)](https://www.eea.europa.eu/data-and-maps/data/world-digital-elevation-model-etopo5)) and were averaged over the study grid.

REFERENCES FOR Supplementary material S1

Balenghien, T., Alexander, N., Arnþórsdóttir, A. L., Bisia, M., Blackwell, A., Bødker, R., … Wint, W. G. R. (2020). VectorNet Data Series 3: Culicoides Abundance Distribution Models for Europe and Surrounding Regions. *Open Health Data*, *7*(1). doi:10.5334/ohd.33

Conte, A., Giovannini, A., Savini, L., Goffredo, M., Calistri, P., & Meiswinkel, R. (2003). The Effect of Climate on the Presence of Culicoides imicola in Italy. *Journal of Veterinary Medicine, Series B*, *50*(3), 139–147. doi:10.1046/j.1439-0450.2003.00632.x

Gilbert, M., Nicolas, G., Cinardi, G., Boeckel, T. P. V., Vanwambeke, S. O., Wint, G. R. W., & Robinson, T. P. (2018). Global distribution data for cattle, buffaloes, horses, sheep, goats, pigs, chickens and ducks in 2010. *Scientific Data*, *5*(1), 180227. doi:10.1038/sdata.2018.227

Karger, D. N., Schmatz, D. R., Dettling, G., & Zimmermann, N. E. (2020). High-resolution monthly precipitation and temperature time series from 2006 to 2100. *Scientific Data*, *7*(1), 248. doi:10.1038/s41597-020-00587-y

Turner, J., Bowers, R. G., & Baylis, M. (2013). Two-Host, Two-Vector Basic Reproduction Ratio (R 0) for Bluetongue. *PLoS ONE*, *8*(1), e53128. doi:10.1371/journal.pone.0053128
