# Supplementary material for: Quantitative risk assessment for the introduction of bluetongue virus into mainland Europe by long‐distance wind dispersal of Culicoides spp.: A case study from Sardinia
Source: Risk Anal. 2024 Jul 2;45(1):108–27. doi: 10.1111/risa.14345 (PMC11735344; doi:10.1111/risa.14345)
Supplement: Supplementary file 2 — SUPPORTING INFORMATION [file RISA-45-108-s003.docx]

**Quantitative risk assessment for the introduction of Bluetongue virus into mainland Europe by long-distance wind dispersal of Culicoides spp. : A case study from Sardinia.**

Supplementary material S2: Sensitivity analysis and uncertainty assessment

# S2.1 Parametrization settings for sensitivity analysis

Input factors kept fixed in the analysis:

| Var Abbr | Description | Fixed value | Rationale |
| --- | --- | --- | --- |
| $\boldsymbol{N}_{\boldsymbol{h}}$ | Host population of type $h$ | $N_{c}$= $N_{s}$ = 1000 | Arbitrary set. $C$ set for Cattle and $S$ for Small Ruminants |
| $\boldsymbol{time}$ | Duration of aerial transport | 1 day | Shortest scenario |
| $\boldsymbol{Pre}\boldsymbol{v}_{\boldsymbol{h}}$ | Disease prevalence in host type $h$ | $Prev_{c}=Prev_{s}$ = 0.01 | Moderate prevalence for both host types |

Input factors tested in the analysis:

| Var Abbr | Description | Domain range and probability distributions | Rationale |
| --- | --- | --- | --- |
| $\boldsymbol{w}\boldsymbol{N}_{\boldsymbol{v}}$ | expected vector abundance of species$v$ | ${wN}_{1}$ = ${wN}_{2}$ ~ Uniform (0, 10^7^) | Upper limit fixed as double of the maximal value of VectorNet data (18.4 log) |
| $\boldsymbol{U}_{\boldsymbol{i}}$ | probability for vector to be uplifted in the air mass | $U_{i}$~ Uniform (0,1) | Maximal range. No empirical data available to reduce the full interval range [0,1] |
| $\boldsymbol{H}_{\boldsymbol{ij}}$ | Hysplit probability of wind dispersal from source$i$ to destination $j$ | $H_{ij}$~ Uniform (0,1) | Maximal range considered |
| $\boldsymbol{a}_{\boldsymbol{v}}$ | biting rate of vector species $v$ | $a_{1}=a_{2}$ ~ Uniform (10^-2^,1) | Equivalent to the inverse of time between 2 blood meals^[1]^; at least 1 bite every 100 days; at most 1 bite per day; assuming same biting rate range for the 2 vector species |
| $\boldsymbol{\omega}_{\boldsymbol{v}}$ | virogenesis rate, i.e rate at which latent vector become infectious for vector species $v$ | $\omega_{1}=\omega_{2}\sim$Uniform (1/50,1/0.04) | Equivalent to 1/EIP (Extrinsic Incubation Period). EIP is highly variable between few days to 40 days^[2]^. Range set between 1 hour and 50 days |
| $\boldsymbol{\mu}_{\boldsymbol{v}}$ | natural mortality rate for vector species $v$ | $\mu_{1}$ ~ Uniform (1/60,1/2)  $\mu_{2}$ ~ Uniform (1/100,1/5) | Equivalent to the inverse of the vector lifespan; between 2 days and 60 days at most for *C.imicola;* between at 5 days and 100 days for Obsoletus complex ^[3]^, assuming higher longevity for this vector^[4]^ |
| $\boldsymbol{\beta}_{\boldsymbol{v}}$ | probability of effective transmission from a host to vector given an effective contact | $\beta_{1}=\beta_{2}$~ Uniform (0, 1) | Equivalent to vector competence. Vector competence showed to be variable among BTV serotypes and vector species. |
| $\boldsymbol{\sigma}_{\boldsymbol{v}}$ | host preference for a vector species $v$ | $\sigma_{v}$~ Uniform (0,1) | sigma<1 means cattle preference; sigma= 1 means no specific host preference ^[5]^ |
| $\boldsymbol{r}_{\boldsymbol{h}}$ | recovery rate in host species $h$ | $r_{c}$ ~ Uniform (1/200,1/6) $r_{s}$~ Uniform (1/250,1/6) | Equivalent to the inverse of the viraemic period in days. Distribution maximums were set to 1/6 days for cattle and small ruminants (viraemia detected by virus isolation)^[6]^. Distribution minimums were set to 1/200 days for cattle and 1/250 days for small ruminants (round up values from 167 days and 222 days of maximum viraemic period detected by PCR, respectively for cattle and sheep ^[6]^) |
| $\boldsymbol{d}_{\boldsymbol{h}}$ | disease induced mortality in species $h$ | $d_{c}$ ~ Uniform (1/1825,0.1) $d_{s}$ ~ Uniform (1/1460,0.75) | Equivalent to the inverse of the host survival time. Lower estimates correspond to 5 years for cattle and 4 years for small ruminants and upper estimates were retrieved form literature ^[5]^ |
| $\boldsymbol{b}$ | probability of effective transmission from a vector to a host given an effective contact. | $b$ ~ Uniform (0,1) | While literature usually reports the interval [0.8,1], an extended interval [0,1] was considered here to account for potential non susceptibility of the host (vaccination or breed effect) |

# S2.2 Individual impact of the 6 most influential parameters on risk outputs


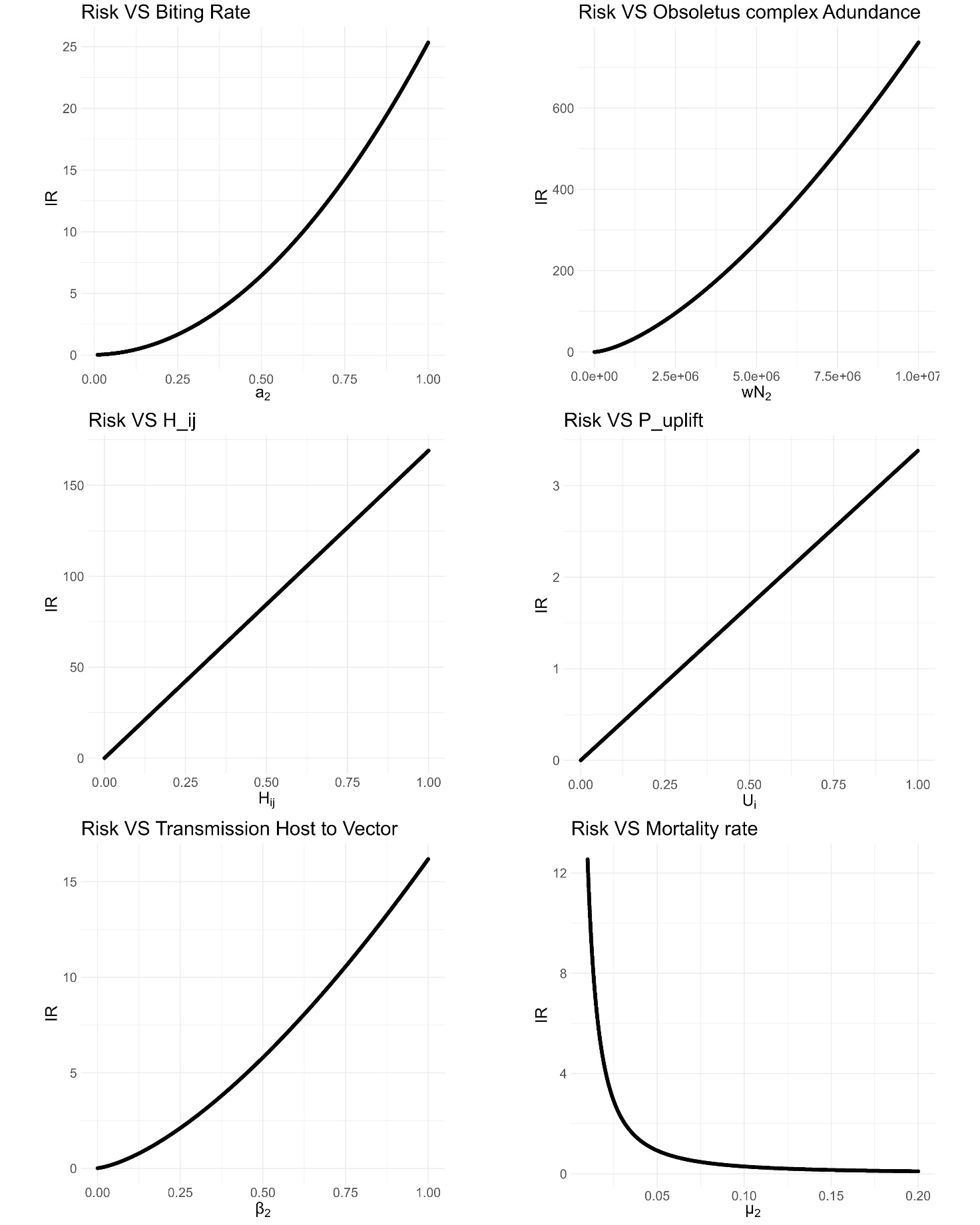


# S2.3 Parametrization settings for uncertainty assessment

Input factors that were not included in the uncertainty assessment were those that, according to the results of the sensitivity analysis, had limited impact in the model outputs: the transmission rate from vector to host ($b)$, the host recovery rates ($r_{c}, r_{s}$), the host mortality rates ($d_{c}, d_{s}$), the host preference ($\sigma_{hv}$), the host abundance ($N_{h}$), and vector virogenesis rate ($\omega_{1},\omega_{2}$). Although identified as an influencing factor, the probability for aerial transportation from source $i$to destination $j$ ($H_{ij}$) was not included because the uncertainty was considered low (based on historical meteorological data, scenario of 24 hours of dispersion, consistency over the 3 years tested)

Variation introduced for each influencing factors:

| Var Abbr | Description | Domain range and probability distributions | Rationale |
| --- | --- | --- | --- |
| $\boldsymbol{N}_{\boldsymbol{v}}$ | Maximal yearly vector abundance species $v$ | $N_{1}$ = $N_{2}$ ~ Normal (mean, sd) | Means and standard deviations (sd) were retrieved from VectorNet data^[7]^. They respectively correspond to the point estimate and the error estimates per grid cell provided by the machine learning algorithm. |
| $\boldsymbol{w}\boldsymbol{N}_{\boldsymbol{v}}$ | expected vector abundance of species$v$ | $w=\frac{e^{z}}{1+ e^{z}}$ with $z = \beta_{0}+ \sum_{k=1}^{K} \beta_{k}x_{k}$ | As $wN_{v}$ depends on the function $z$, variation was introduced on each coefficient of the equation published in literature ^[8]^. It was assumed that each coefficient followed a normal distribution between its mean estimate and its standard deviations (See specific number in Supplementary S1.2) |
| $\boldsymbol{U}_{\boldsymbol{i}}$ | probability for vector to be uplifted in the air mass | $U_{i}$~ Uniform (10^-3^,0.5) | The upper value used by Hall et al^.[61]^ was fixed as the minimal value. Maximal limit was arbitrary fixed at 50%. |
| $\boldsymbol{a}_{\boldsymbol{v}}$ | daily biting rate of vector species $v$ | $a_{v}$ ~ Uniform (Point estimate – ‘$noise’$, Point estimate + ‘$noise’$) | $a_{v}$ is a function of temperature as described in main text (figure 1). Variation around each point estimate was added. ‘$noise'$ represents here 10% of the point estimate |
| $\boldsymbol{\mu}_{\boldsymbol{v}}$ | natural mortality rate in vector species $v$ | $\mu_{v}$~ Uniform (Point estimate – $‘noise’$, Point estimate + $‘noise’$) | $\mu_{v}$ is a function of temperature as described in main text (figure 1). Variation around each point estimate was added. ‘$noise'$ represents here 10% of the point estimate |
| $\boldsymbol{\beta}_{\boldsymbol{v}}$ | probability of effective transmission from a host to vector given an effective contact | $\beta_{v}$~ Uniform (Point estimate – $‘noise’$, Point estimate + $‘noise’$) | $\beta_{v}$ is a function of temperature as described in main text (figure 1). Variation around each point estimate was added. ‘$noise'$ represents here 10% of the point estimate |

REFERENCES for Supplementary material S2

1. Mullens, B. A., Gerry, A. C., Lysyk, T. J., & Schmidtmann, E. T. (2004). Environmental effects on vector competence and virogenesis of bluetongue virus in Culicoides: interpreting laboratory data in a field context. *Veterinaria Italiana*, *40*(3), 160–166.

2. Braks, M., Mancini, G., & Goffredo, M. (2017). Risk of vector‐borne diseases for the EU: Entomological aspects – Part 1. *EFSA Supporting Publications*, *14*(2). <https://doi.org/10.2903/sp.efsa.2017.en-1173>

3. Goffredo, M., Romeo, G., Monaco, F., Gennaro, A. D., & Savini, G. (2004). Laboratory survival and blood feeding response of wild-caught Culicoides obsoletus Complex (Diptera: Ceratopogonidae) through natural and artificial membranes. *Veterinaria Italiana*, *40*(3), 282–285.

4. Meiswinkel, R., Baldet, T., Deken, R. de, Takken, W., Delécolle, J.-C., & Mellor, P. S. (2008). The 2006 outbreak of bluetongue in northern Europe—The entomological perspective. *Preventive Veterinary Medicine*, *87*(1–2), 55–63. <https://doi.org/10.1016/j.prevetmed.2008.06.005>

5. Hartemink, N. A., Purse, B. V., Meiswinkel, R., Brown, H. E., Koeijer, A. de, Elbers, A. R. W., Boender, G.-J., Rogers, D. J., & Heesterbeek, J. A. P. (2009). Mapping the basic reproduction number (R0) for vector-borne diseases: A case study on bluetongue virus. *Epidemics*, *1*(3), 153–161. <https://doi.org/10.1016/j.epidem.2009.05.004>

6. Dórea, F. C., Swanenburg, M., Roermund, H., Horigan, V., Vos, C., Gale, P., Lilja, T., Comin, A., Bahuon, C., Zientara, S., Young, B., Vial, F., Kosmider, R., & Lindberg, A. (2017). Data collection for risk assessments on animal health (Acronym: DACRAH) : Final Report. *EFSA Supporting Publications*, *14*(1), 1171E. <https://doi.org/10.2903/sp.efsa.2017.en-1171>

7. Balenghien, T., Alexander, N., Arnþórsdóttir, A. L., Bisia, M., Blackwell, A., Bødker, R., Bourquia, M., Boutsini, S., Carpenter, S., Colenutt, C., Culverwell, L., Cvetkovikj, A., Dascălu, L., Regge, N. D., Dhollander, S., Elbers, A., England, M., Filatov, S., Garros, C., … Wint, W. G. R. (2020). VectorNet Data Series 3: Culicoides Abundance Distribution Models for Europe and Surrounding Regions. *Open Health Data*, *7*(1). <https://doi.org/10.5334/ohd.33>

8. Conte, A., Giovannini, A., Savini, L., Goffredo, M., Calistri, P., & Meiswinkel, R. (2003). The Effect of Climate on the Presence of Culicoides imicola in Italy. *Journal of Veterinary Medicine, Series B*, *50*(3), 139–147. <https://doi.org/10.1046/j.1439-0450.2003.00632.x>
