# Supplementary material for: Quantitative risk assessment for the introduction of bluetongue virus into mainland Europe by long‐distance wind dispersal of Culicoides spp.: A case study from Sardinia
Source: Risk Anal. 2024 Jul 2;45(1):108–27. doi: 10.1111/risa.14345 (PMC11735344; doi:10.1111/risa.14345)

**Quantitative risk assessment for the introduction of Bluetongue virus into mainland Europe by long-distance wind dispersal of Culicoides spp. : A case study from Sardinia.**

Supplementary material S3: Additional Figures

**Fig. S3.1 : Maximal annual abundance of *C. imicola* (left) and Obsoletus Complex (right) in Europe retrieved from VectorNet database and computed at grid resolution 0.5° (expressed in number of individuals). Insert in both Fig.s shows the spatial distribution of the maximal annual abundance of each species of *Culicoides* in Sardinia. The blue contour areas in inserts indicate areas considered as source in the model.**


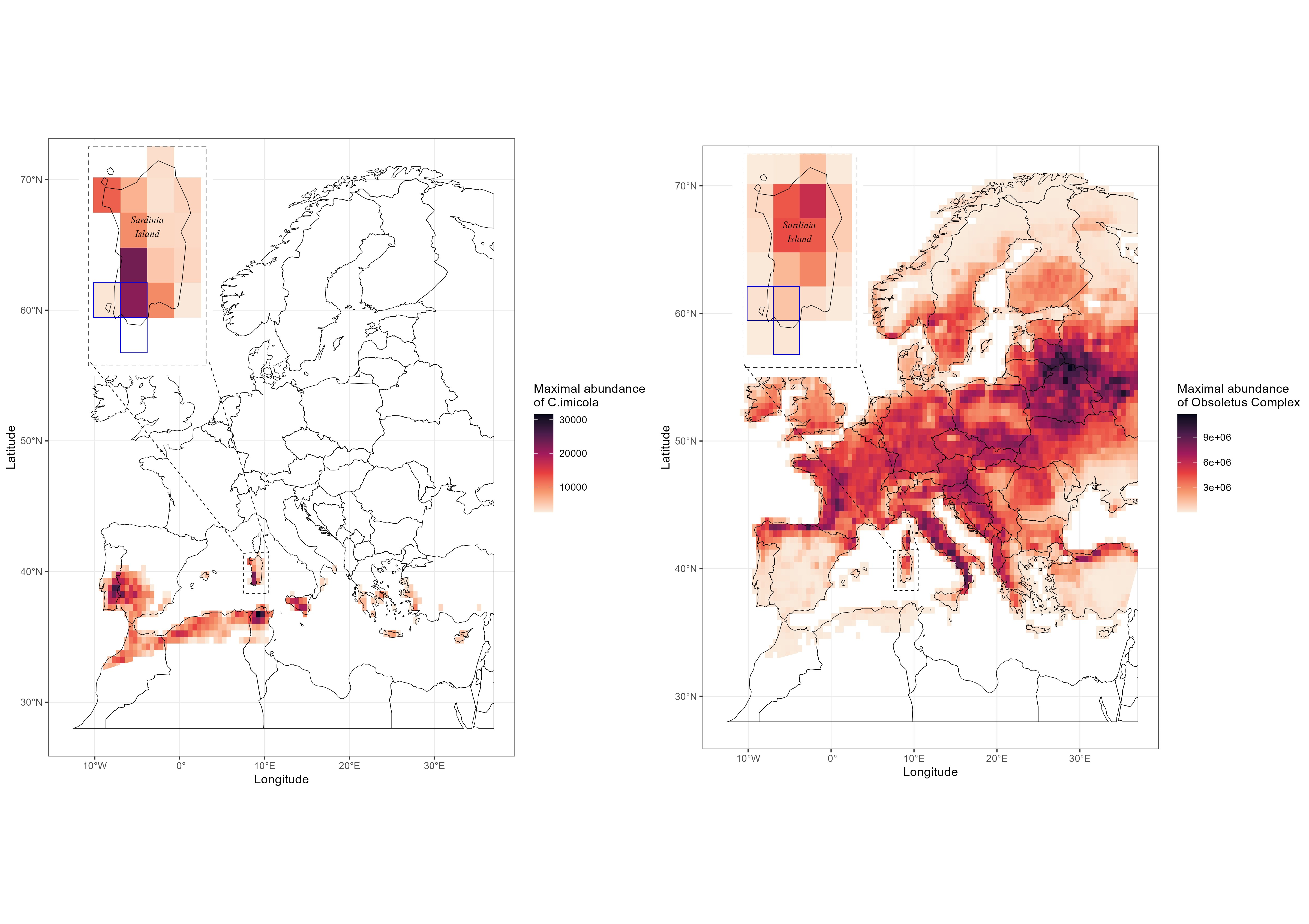


**Fig. S3.2: Spatial distribution in the abundance of cattle (left) and small ruminants (right) in Europe. Here, estimates of abundance, expressed in number of heads, are retrieved from the Livestock Grid of the World database and aggregated at grid resolution 0.5°.**


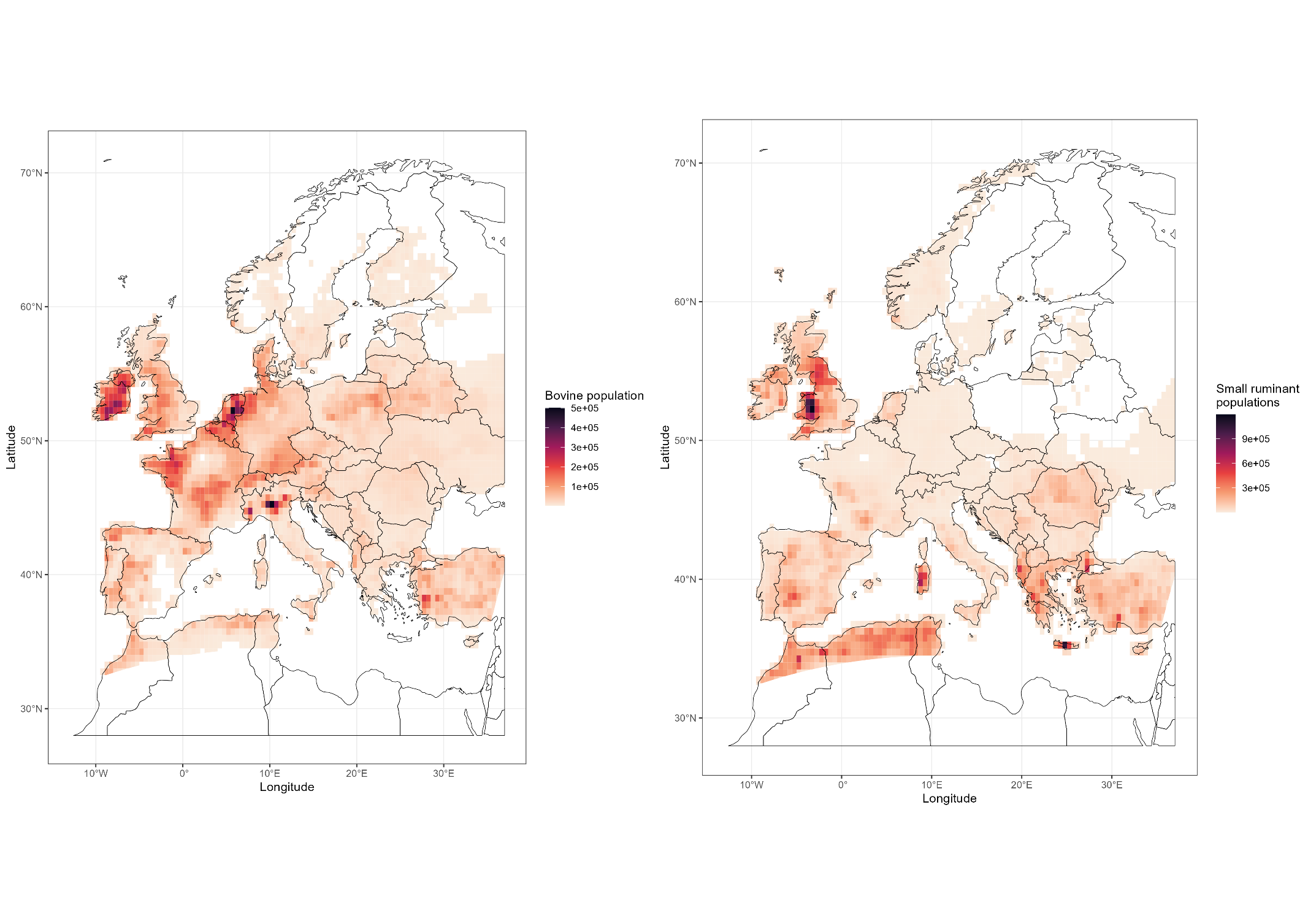


**Fig. S3.3: Spatial distribution of the overall mean probability of long-distance wind dispersal from southwestern Sardinia to any destination in Europe, considering 48-h maximal flight duration. Here, overall mean probability** ${\bar{\boldsymbol{P}}}_{\boldsymbol{ij}}$ **is computed over the 36 weeks of the study period.**


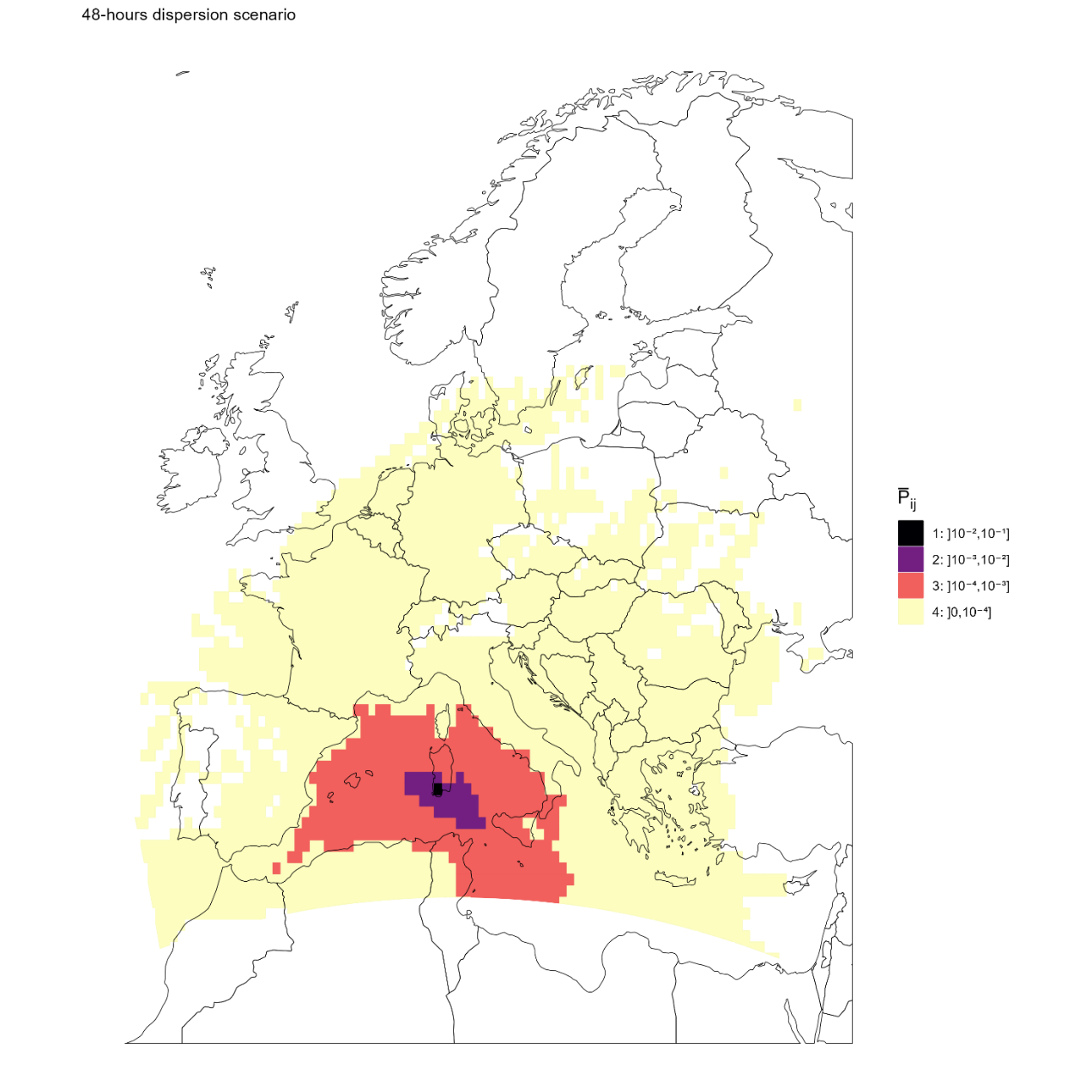


**Fig. S3.4:** **Spatial distribution of the mean probability of long-distance wind dispersal from southwestern Sardinia, considering 48-h maximal flight duration. Here, mean probability** $\boldsymbol{P}_{\boldsymbol{ij}}$ **is computed for each week of the study period considered constant environmental conditions.**


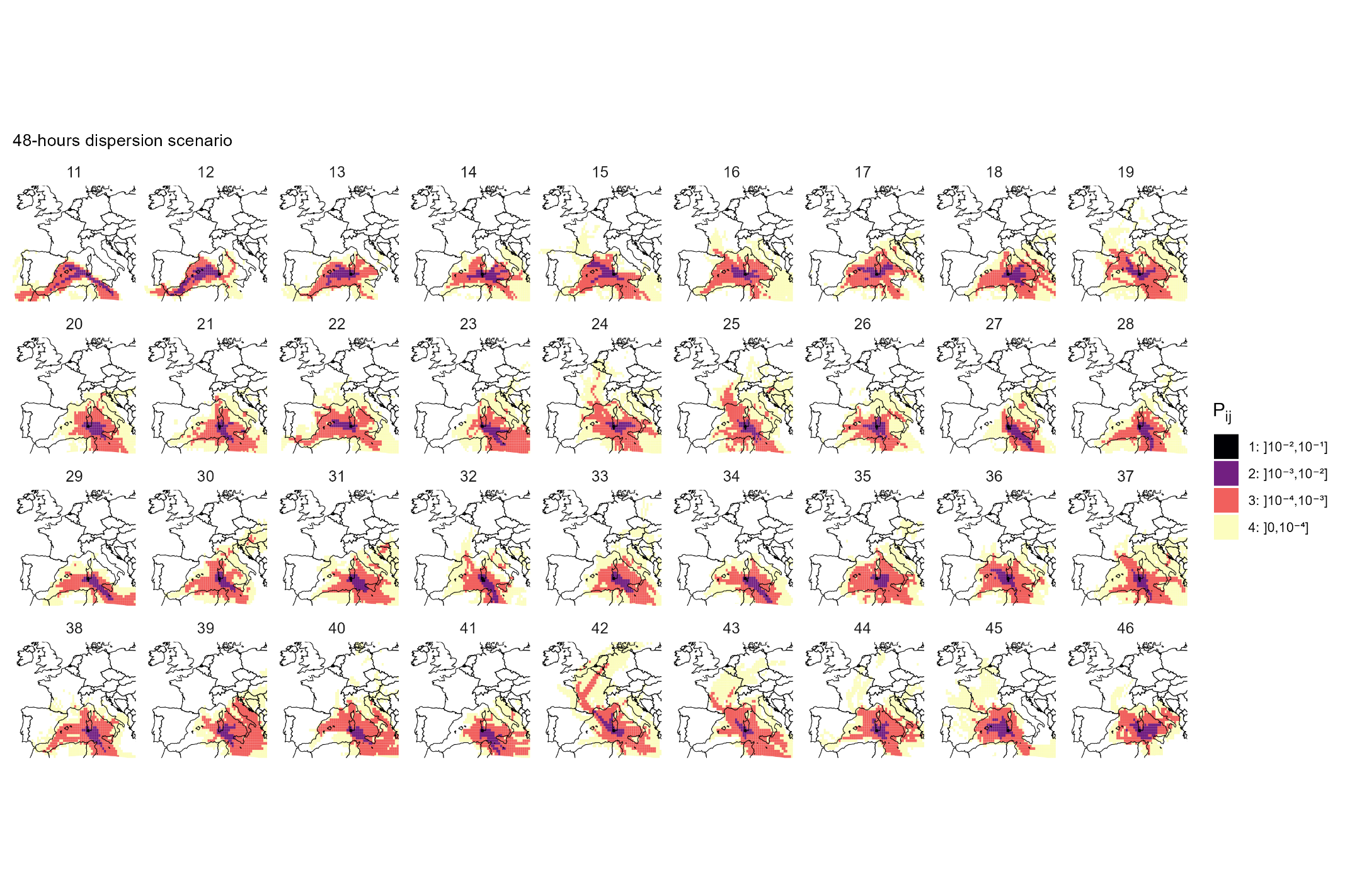


**Fig. S3.5: Spatial distribution of** ${\boldsymbol{R}_{\mathbf{0}}}_{\boldsymbol{j}}$ **of BTV in Europe considering 2 host types (cattle and small ruminants) and 2 vector populations (*C. imicola* and Obsoletus complex). Here, the values of** ${\boldsymbol{R}_{\mathbf{0}}}_{\boldsymbol{j}}$ **in each grid cell *j* is computed for each week of the study period and considering constant weekly environmental conditions.**


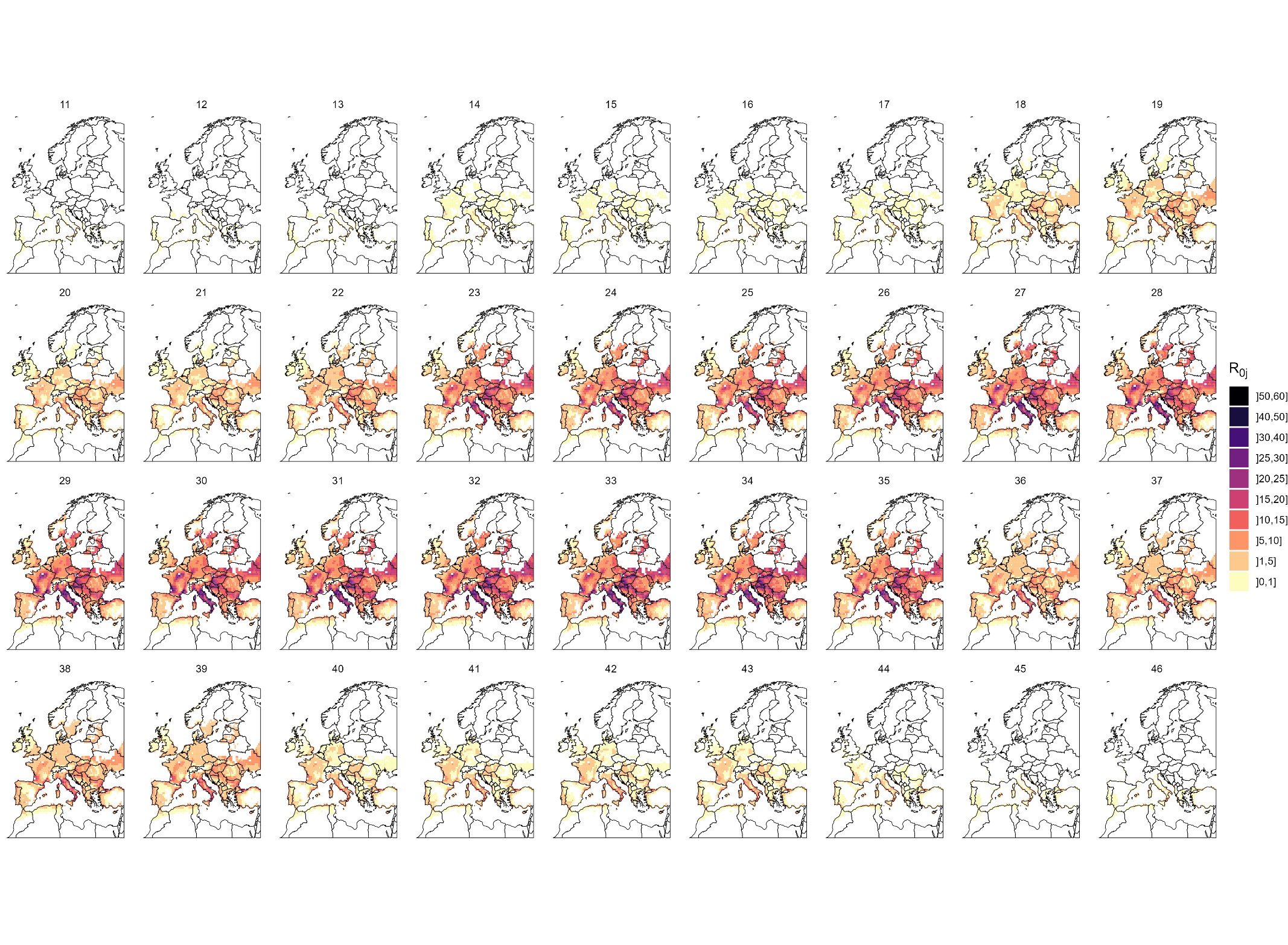


**Fig. S3.6: Spatial distribution of** $\sqrt{\boldsymbol{R}_{\boldsymbol{1}\boldsymbol{1}_{\boldsymbol{j}}}}$ **of BTV in Europe considering 2 host types (cattle and small ruminants) and only *C. imicola* as vector. Here, the values of** $\sqrt{\boldsymbol{R}_{\boldsymbol{1}\boldsymbol{1}_{\boldsymbol{j}}}}$ **in each grid cell *j* is computed for each week of the study period and considering constant weekly environmental conditions.**
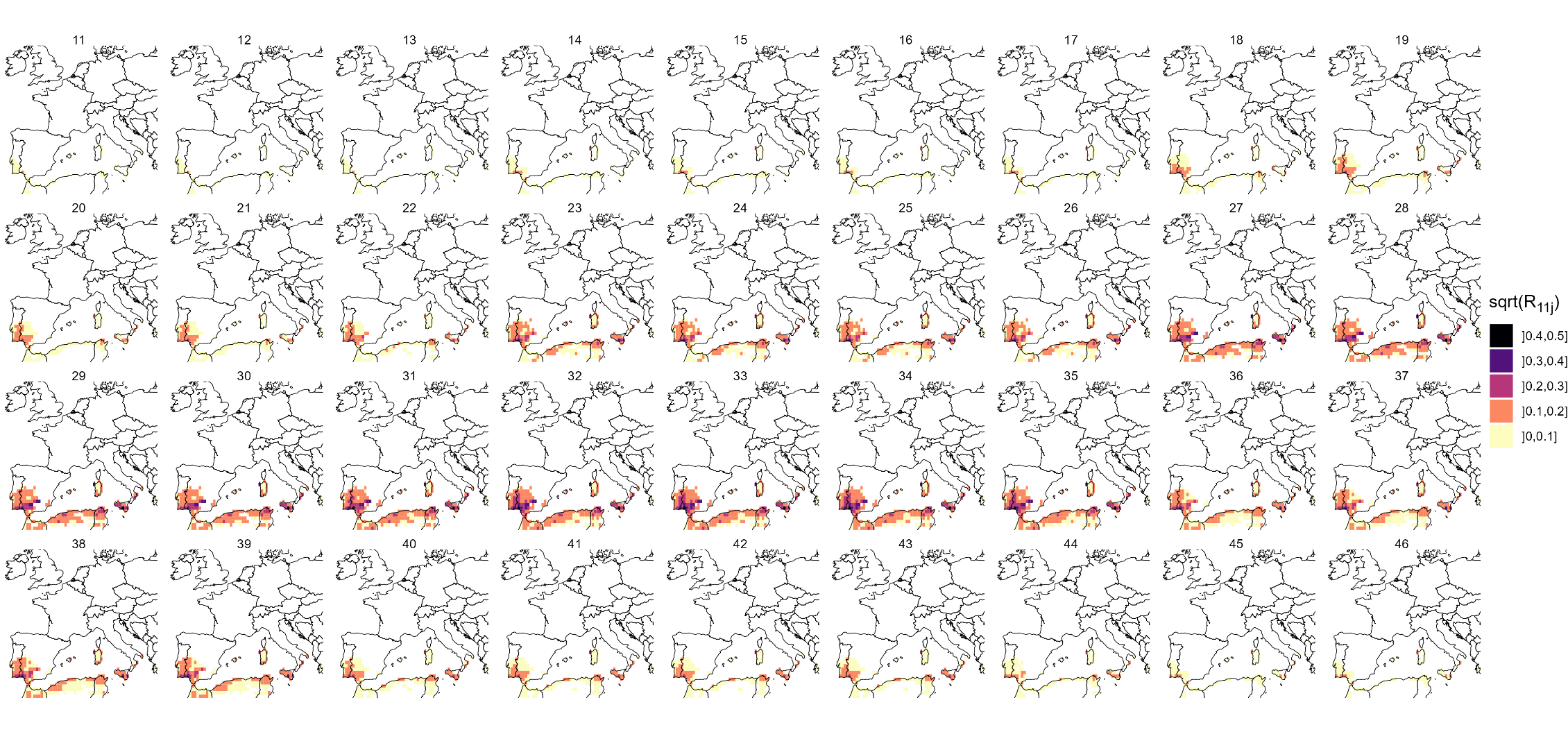


**Fig. S3.7: Spatial distribution of the overall mean risk of introduction from southwestern Sardinia to any destination in Europe, considering *C. imicola* is the only competent vector for disease transmission and different maximal flight duration (24-h and 48-h scenarios). Here, overall mean risk of introduction** $\bar{\boldsymbol{IR}}$ **is computed over the 36 weeks of the study period.**


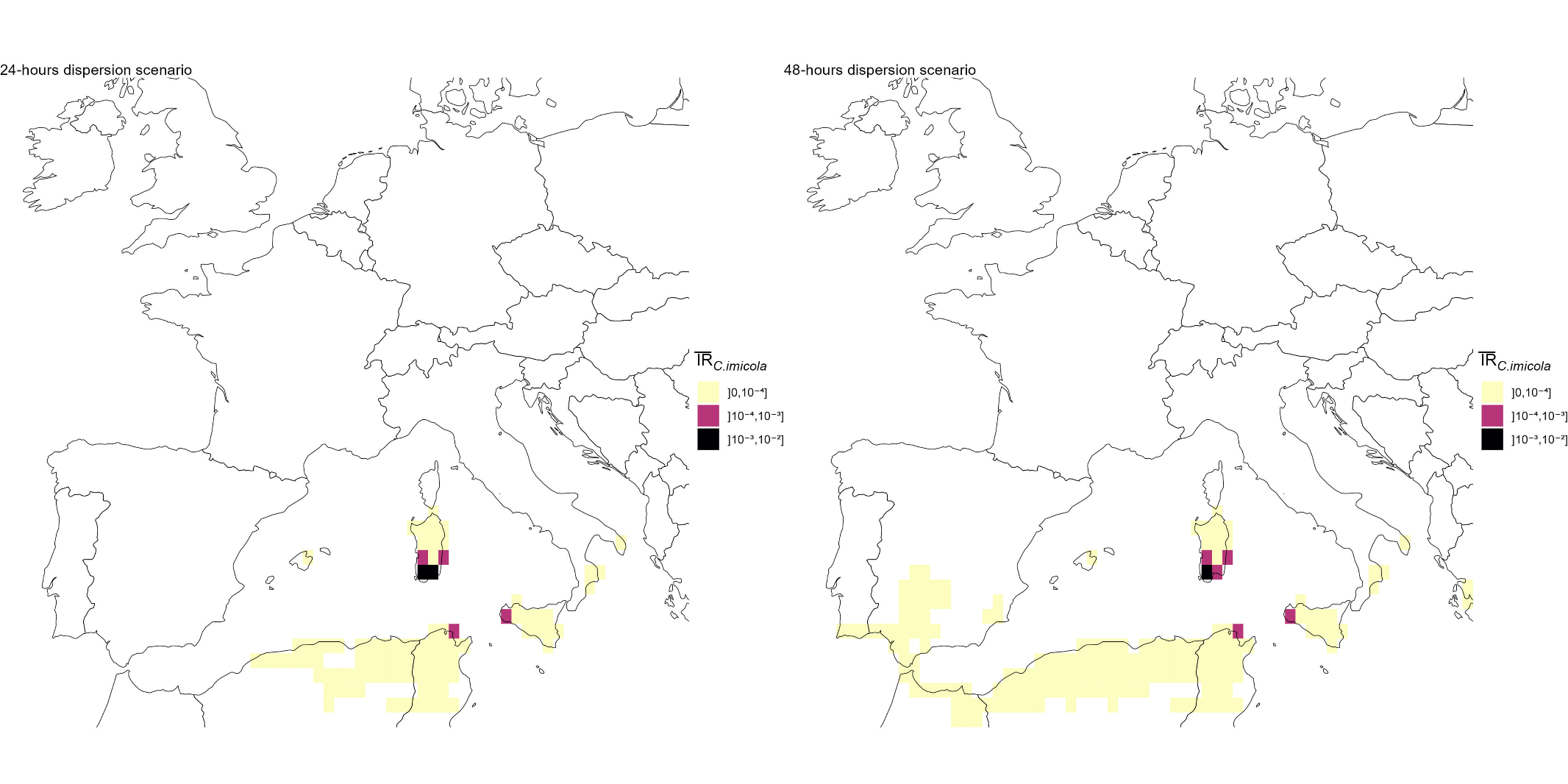


**Fig. S3.8: Spatial distribution of the mean risk of introduction from southwestern Sardinia to any destination in Europe in each week of the study period and considering a 24-h maximum flight duration of vectors. Here, the mean risk of introduction** $\boldsymbol{IR}$ **was computed considering constant weekly environmental conditions.**


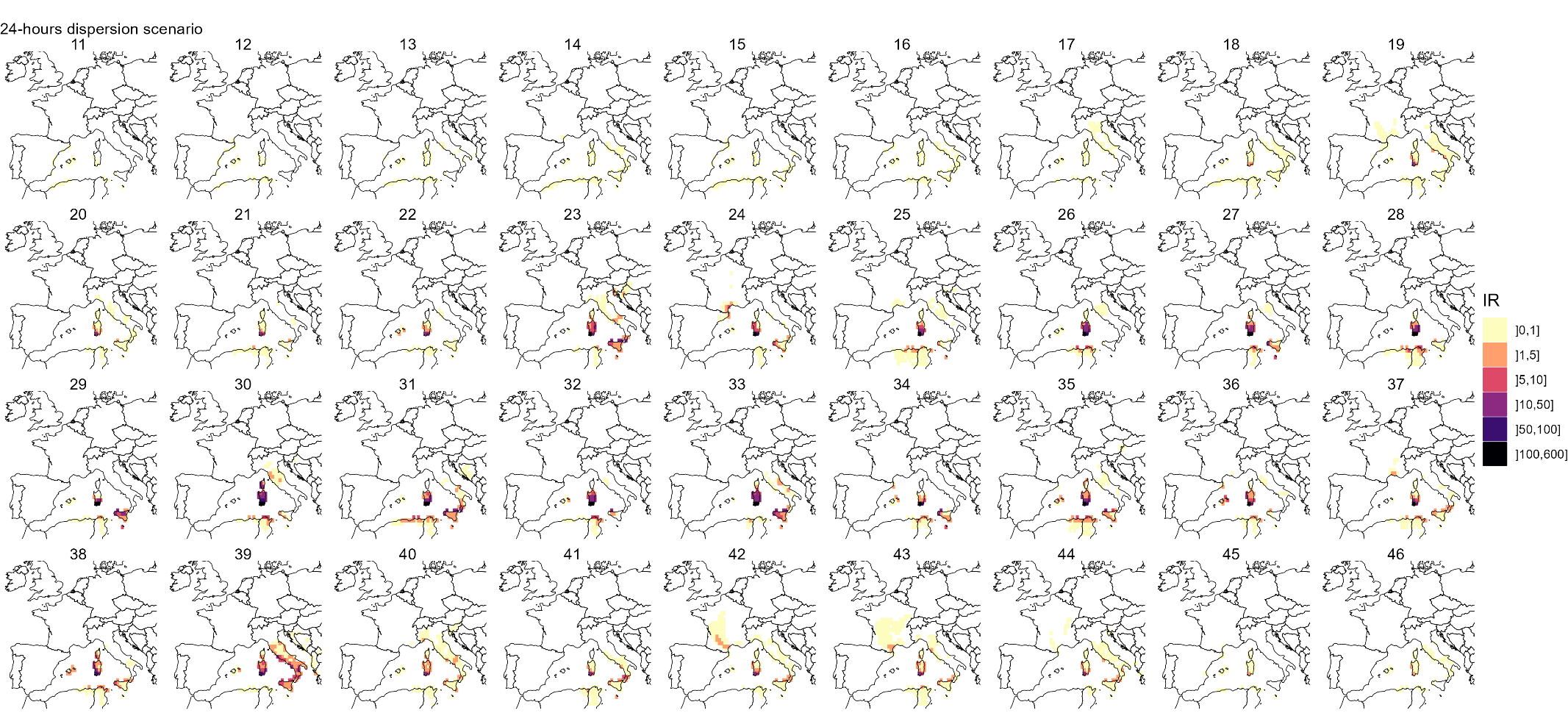


**Fig. S3.9: Spatial distribution of the mean risk of introduction from southwestern Sardinia to any destination in Europe in each week of the study period and considering a 48-h maximum flight duration of vectors. Here, the mean risk of introduction** $\boldsymbol{IR}$ **was computed considering constant weekly environmental conditions.**


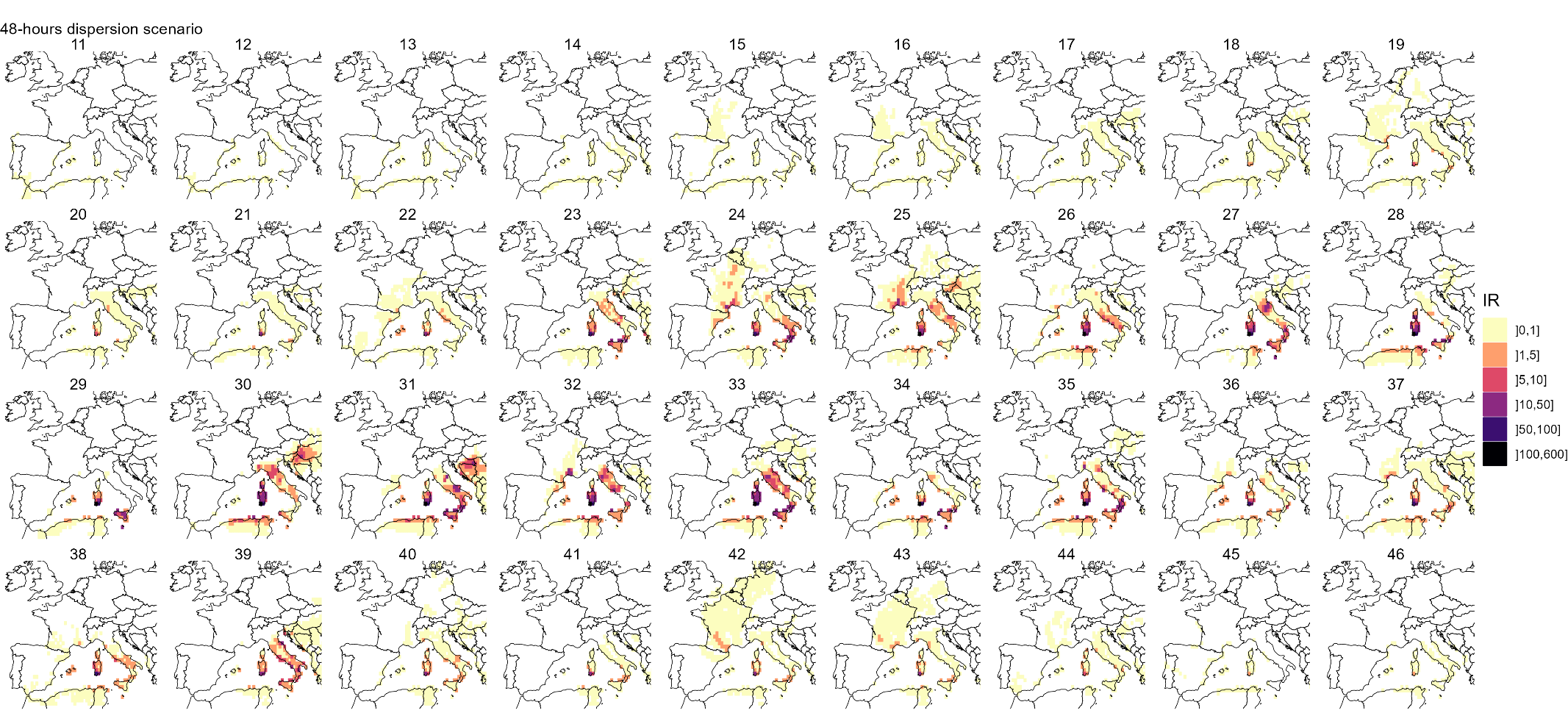


**Fig. S3.10: Spatial distribution of the overall risk of introduction from southwestern Sardinia to any destination in Europe, considering a 48-h maximum flight duration of vectors. Here, the risk of introduction was expressed as (A) the overall risk of introduction (**$\bar{\boldsymbol{IR}}$**) in Europe and (B) the number of weeks in which the mean risk of introduction** $\boldsymbol{IR}$ **is greater than 1 (**$\boldsymbol{W}_{\boldsymbol{IR>1}}$**). Here,** **the mean risk of introduction** $\boldsymbol{IR}$ **was computed considering constant weekly environmental conditions and a 48-h maximum flight duration of vectors.**

**(B)**

**(A)**


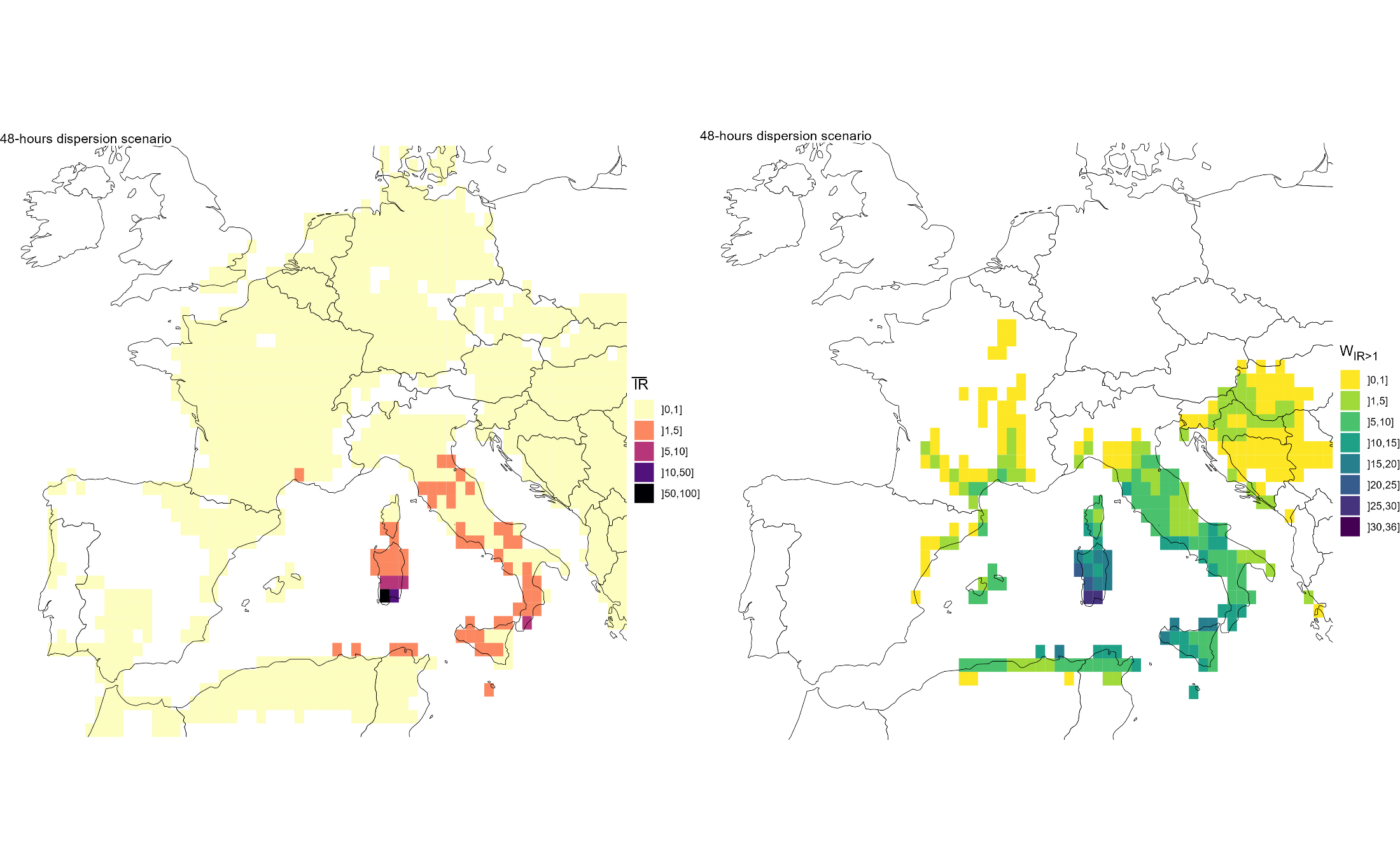


**Fig. S3.11: Uncertainty in the estimated mean risk of introduction from southwestern Sardinia to any destination in Europe. Uncertainty is measured as the probability (**$\boldsymbol{P}_{\boldsymbol{IR>1}}$**) for which the estimate of** $\boldsymbol{IR}$ **is greater than 1, considering uncertainty around key parameters. Here, estimates of IR was computed for each week of the study period and considering constant weekly environmental conditions and a 24-h maximum flight duration of vectors.**


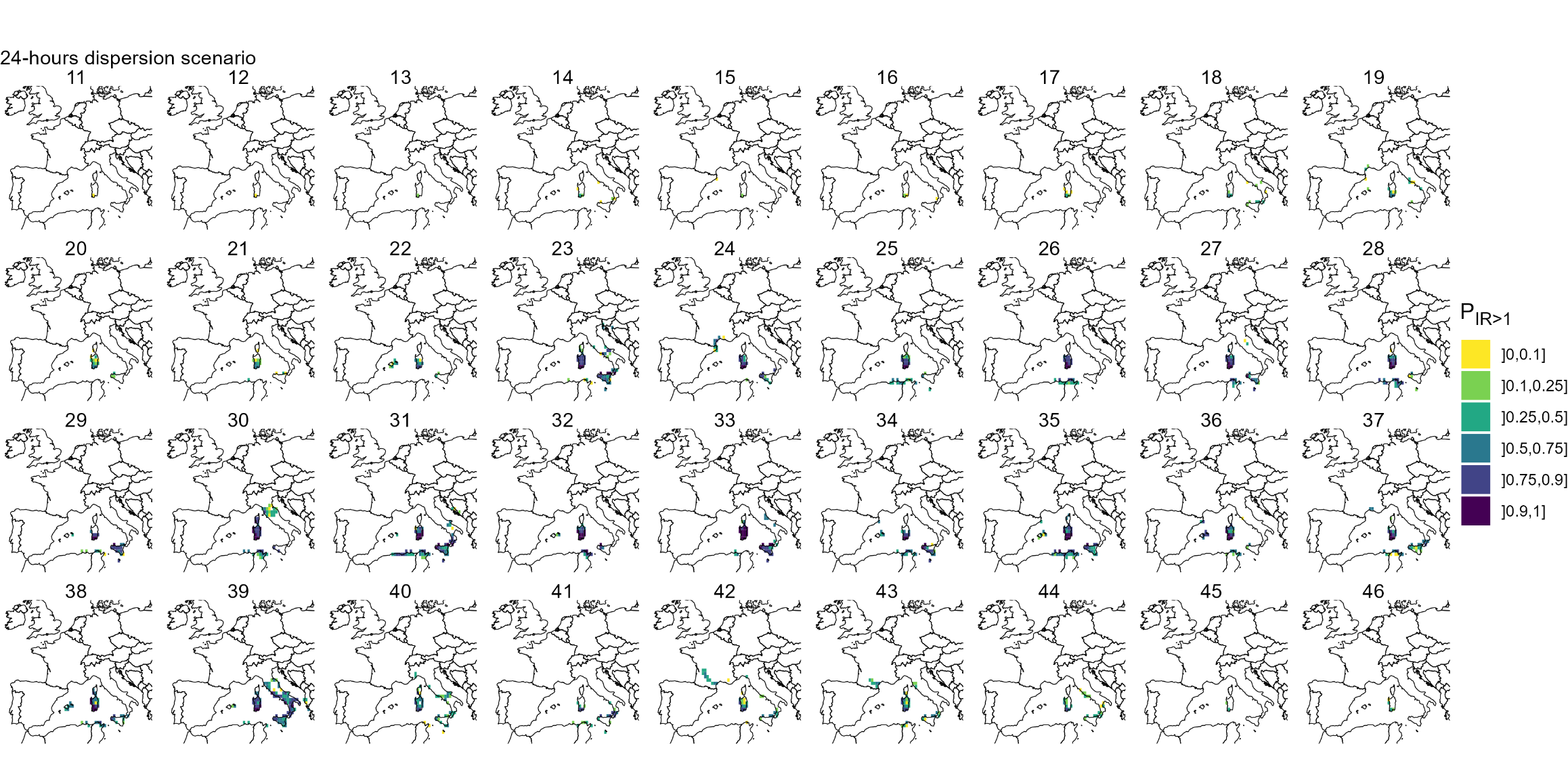

Supplement: Supplementary file 3 — SUPPORTING INFORMATION [file RISA-45-108-s002.docx]
